# Impact of the diet in the gut microbiota after an inter-species microbial transplantation in fish

**DOI:** 10.1038/s41598-024-54519-6

**Published:** 2024-02-18

**Authors:** Alberto Ruiz, Enric Gisbert, Karl B. Andree

**Affiliations:** Aquaculture Program, Institut de Recerca i Tecnologia Agroalimentàries (IRTA), Centre de La Ràpita, Crta. Poble Nou, km 5.5, 43540 La Ràpita, Spain

**Keywords:** Food microbiology, Antimicrobials, Applied microbiology, Microbiome, Bacterial development, Bacterial host response, Marine microbiology

## Abstract

Inter-species microbial transplantations offer the possibility of transferring species-specific microbes and their associated functionality. As a conceptual approach, an intestinal microbiota transplant (IMT) between two marine carnivorous fish species that thrive in different environmental conditions was conducted: from donor Atlantic salmon (*Salmo salar*) to recipient gilthead seabream (*Sparus aurata*), after obliterating its basal microbiota with an antibiotic treatment. To confirm that the gut microbiota was able to recover after antibiotics without the influence of the diet, a group of gilthead seabream not submitted to the IMT was kept fasted as an internal control. To assess the effect of the diet after the IMT, two groups of gilthead seabream were respectively fed with their typical diet and with Atlantic salmon diet. At 36 days post-IMT, the gut of the individuals fed with their typical diet was dominated by the feed-associated bacteria, while those fed with the salmon diet had developed a unique microbiota from the convergence of the diet, donor, and recipient microbiota. These results suggested that an intestinal microbiota transplantation may be effective if the basal microbiota from the gut is first cleared and a targeted dietary modification is provided to maintain and enrich the novel bacteria species over time.

## Introduction

The gastrointestinal tract is one of the most densely populated ecosystems on our planet where the microbial populations inhabiting it have developed tight relationships with the host over millions of years of co-evolution^1^, constituting what is known by many as a holobiont. In this sense, the intestinal microbiota has a major impact on the host health through a wide range of functions, such as feed digestion, nutrient metabolism, energy homeostasis, immune system modulation, barrier function and mucosal integrity, among others^2^. Moreover, an impairment in microbial composition or in the host-microbe interactions (dysbiosis) can lead to digestive and systemic imbalances and diseases^2,3^. These host-microbiota interactions are reciprocal. For instance, the bacterial community can stimulate the immune system by their pathogen-associated molecular patterns (PAMPs) that are recognized through pattern recognition receptors (PRRs) which subsequently activate immune signaling pathways; and in turn the host shapes the microbial composition, regulating the abundance of some bacterial communities considered as potential pathogens^4^. Basic to this, it may be thought that the microbiota of any animal is well-tuned to the host species, but this is not an inviolably established fact. Recent studies differentiate between the core microbiota, which is the one comprising stable, permanent, and usually highly abundant members, which persist regardless time and changing factors, and the non-core microbiota, which is transient and modulable^5^. Thus, understanding the intricate relationships between the gut microbiota and the host and their modulation may provide opportunities to promote and ensure a healthy microbiota as well as evaluate its beneficial effects to the host.

Apart from specific and non-specific host factors, several elements can influence the gut microbiota in humans and animals, such as the environmental conditions (i.e., pH, oxygen, temperatures), the age, the diet, the host’s habits, such as physical activity, the presence of diseases and the treatments of those diseases^6–8^. In animal production, as a result of the necessity to reduce antibiotic use, several alternatives to improve animal health through gut microbial modulation have been tested and/or consolidated, such as the application of quorum quenching, anti-microbial peptides, feed additives, among other supplements, including exogenous enzymes, probiotics, and prebiotics, alone or in combination (i.e., synbiotics)^9^. In addition to the above-mentioned strategies, fecal microbiota transplants (FMT) have recently gained attention, as it has the advantage of being a persistent strategy that does not require repeated supply or application^6^, and the advent of metagenomic analyses has given to the field more rigor. Numerous applications in humans and animals involving this strategy have been developed over the last decades^1^. The more common and historical use of FMT is for the establishment of a healthy microbiota into a diseased individual to improve the host health. For instance, human FMT is a well-proven effective treatment against *Clostridium difficile* infections^10^ and clinical trials have also shown promising advances in the treatment of inflammatory bowel diseases, metabolic and neurological disorders, as well as autoimmune diseases^11^. Apart from its therapeutic use in humans, FMT has also been extensively tested with production purposes in the livestock industry. For instance, ruminal transfaunation (transplant of the rumen microbial content) is commonly used to restore digestive and metabolic disorders, and to improve milk production in ruminants^12^. Likewise, inter-species transfaunation of the rumen from bison to cattle have shown to enhance protein digestibility^13^. On the other hand, such successful results have not always been obtained for the swine and poultry industry^1^. In this sense, the fact that the target of the FMT are the stool-associated microorganisms, which are mainly the large-intestinal microbes, rather than the whole-intestinal microbiota, may raise the question “to what extent is FMT effective?”^14^. Fortunately, the ease of handling smaller animals, such as fish, allows the collection of all the microbiota found in the intestine rather than just that from the feces, and as such may be more accurately termed as “Intestinal Microbiota Transplant (IMT)”.

To date, some progress has been made regarding gut microbial transplants in teleosts. In particular, recent assays in African turquoise killifish (*Nothobranchius furzeri*), zebrafish (*Danio rerio*), and large yellow croaker (*Larimichthys crocea*), have shown promising results in IMT and FMT as successful strategies to improve host’s longevity, growth and reproductive performance, digestive capacities, intestinal health, endocrine resilience against exposure to environmental pollutants, and gut microbial diversity, among others^15–17^. Similarly, the work of Legrand et al.^18^ in yellowtail kingfish (*Seriola lalandi*) has shed light on the short-term modulation of the gut microbiota by FMT after an antibiotic treatment. Furthermore, zebrafish has been proposed as a model organism to be colonized by bacteria from human feces to study the interactions between the human and zebrafish microbiota^19,20^, even though few taxa were established in the recipient gut. Analogously, when transplanting mouse microbiota into germ-free zebrafish, although the observed phyla resembled those from the donor, their relative abundances were more similar to those of the recipient individuals before transplantation^21^. The former authors stated that these differences in microbial composition were partly imposed by the differential pressures inherent to host-specific gut habitats^21^. This hypothesis was associated to the fact that the above-mentioned vertebrate species have a divergent evolutionary development among others. However, to date there are no works studying the effect from performing an IMT between two different fish species that naturally thrive in different environmental conditions on the gut bacterial communities.

As a conceptual approach, the present study aimed to provide insight into the influence of host and donor gut bacterial communities by carrying out an inter-species IMT using as models two important aquaculture marine fish species^22^ that are carnivorous, but they grow at different temperatures: the Atlantic salmon (*Salmo salar*) and the gilthead seabream (*Sparus aurata*). Furthermore, this study also aimed to elucidate the impact of diet in the phylogenetic flux and dynamics of the microbiota over time in terms of bacterial diversity, structure, and composition.

## Methods

### Fish husbandry and diets

Under current experimental conditions, Atlantic salmon were used as donors of intestinal microbiota, whereas gilthead seabream were chosen as recipients. In particular, Atlantic salmon with an initial body weight (BW_i_) of 55.0 ± 0.1 g (mean ± SD) were purchased from SARL SALMO (Gonneville-Le Theil, France), while gilthead seabream with BW_i_ = 100.2 ± 0.9 g were obtained from Niordseas S.L. (Villajoyosa, Spain), and both were transported by road to the facilities of IRTA, La Ràpita (Tarragona, Spain). Atlantic salmon parrs were smoltified as described in Salomón et al.^23^. Then, salmon smolts were placed in 2000 L-tanks connected to a water recirculation system (IRTAmar™, Spain) and maintained at a water temperature, pH (pH meter 507, Crison Instruments, Barcelona, Spain) and dissolved oxygen (OXI330, Crison Instruments) of 12.1 ± 0.2 °C, 7.4 ± 0.3, and 9.5 ± 0.2 mg/L respectively, under natural photoperiod (8 h light: 16 h darkness) until the beginning of the experiment. Gilthead seabreams were placed in 2000 L-tanks connected to an IRTAmar™ system and water quality parameters were kept at 19.9 ± 2.3 °C, 7.6 ± 0.4, and 6.3 ± 0.6 mg/L. During this period, fish were fed ad libitum with two different feeds; in particular, Atlantic salmon were fed with an experimental diet containing 40% crude protein, 22% crude fat, and 21.6 MJ/kg digestible energy (2–3 mm pellet size; salmon diet)^23^, whereas gilthead seabream were fed with an experimental compound feed (3–5 mm pellet size) containing 44% crude protein, 20% lipids, and 18 MJ/kg digestible energy (GSB diet).

### Antimicrobial treatment and establishment of baseline criteria

A mixture of antimicrobials (AMs) containing Vancomycin (0.02 g/L), Metronidazole (1.0 g/L), Neomycin (1.0 g/L) and Ampicillin (1.0 g/L) was prepared according to Smith et al.^15^, although the concentration for each antibiotic was doubled to compensate for the larger sized fish used herein. The aim of supplying this antimicrobial cocktail to the recipient gilthead seabream prior to the IMT was for eliminating/reducing the host-resident bacterial communities.

**Figure 1:**
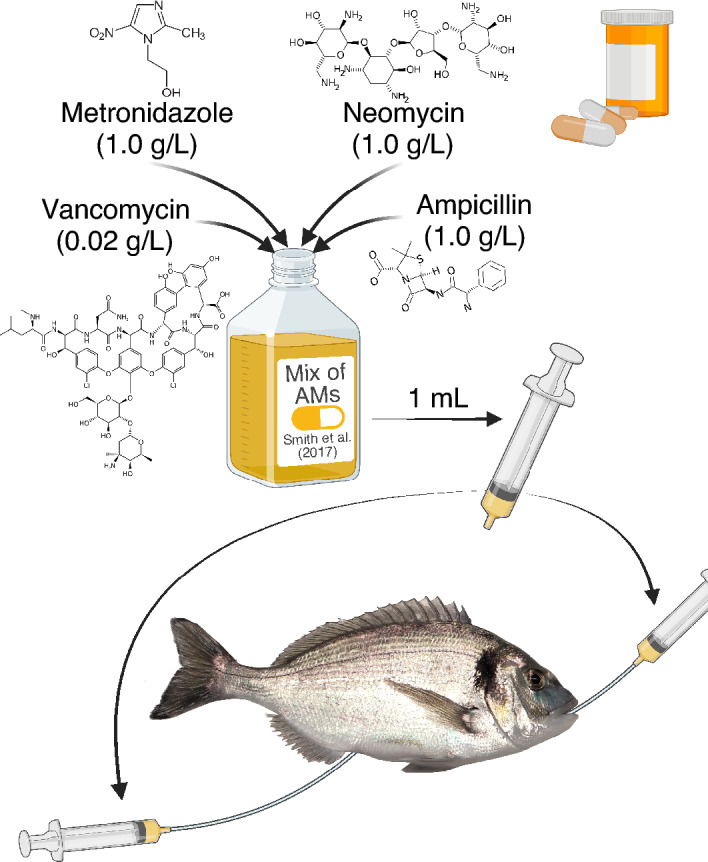
Schematic representation of the composition and administration of the antimicrobial mixture to recipient gilthead seabream (*Sparus aurata*). Created with BioRender.com.

**Figure 2:**
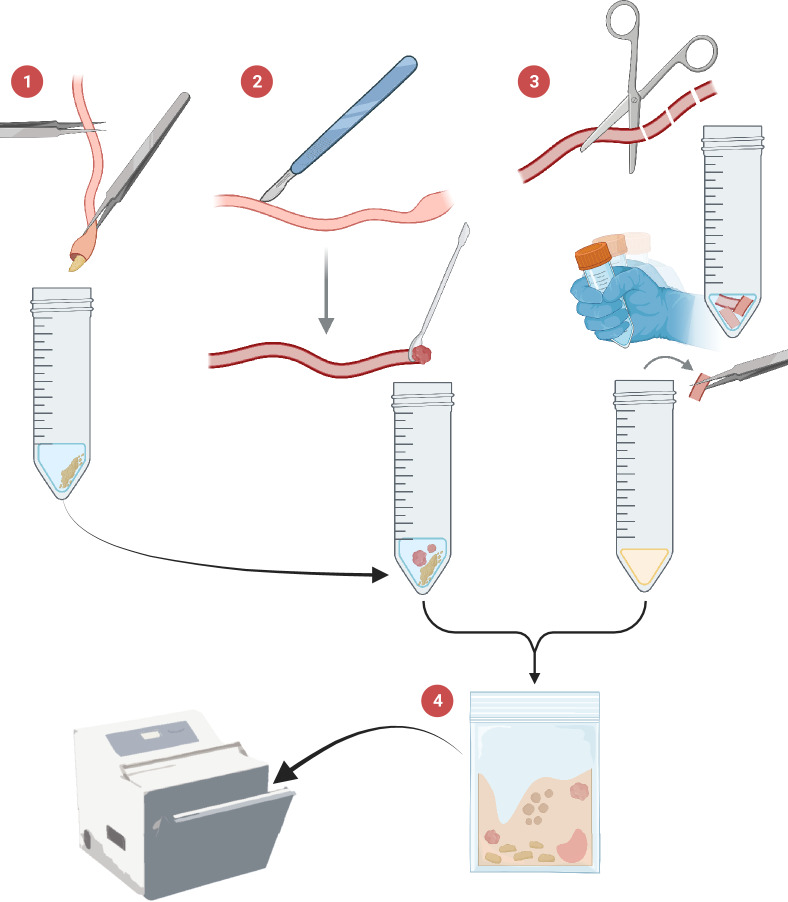
Schematic representation of the steps followed for intestinal sampling of bacteria: 1, stripping for chyme collection; 2, scraping for collection of mucosal content; 3, gathering of bacteria still associated to the tissue by shaking; and 4, pooling and homogenization in a Stomacher. Created with BioRender.com.

To decide the time after the AM treatment at which the intestinal microbiota from the donor salmon should be transplanted into the recipient gilthead seabream the recovery time of the gut-bacterial communities from gilthead seabream after the AMs was evaluated by their growth in TSA (Trypticase Soy Agar) + 0.6% NaCl culture media. Furthermore, this brief assay confirmed the effectiveness of the AM cocktail for elimination of the resident microbiota of the donor fish. For that purpose, three gilthead seabream that had been fasted overnight were hand-netted and anesthetized with buffered tricaine methanesulfonate (MS-222, Sigma-Aldrich, Spain; 100 mg/L). Then, an aliquot of 1 mL of the mixture of AMs was administered by both anal and oral gavage using a 5 mL syringe connected to a cannula (ø = 1.7 mm; ref. 340–6402, Izasa, Spain) (Fig. 1). Orally, the cannula was inserted until the stomach, while anal insertion was *ca.* 2–3 cm towards anterior direction, reaching the mid-posterior intestinal region. After 24 h, the three fish were euthanized in a bucket of water containing an overdose of buffered tricaine methanesulfonate (MS-222, Sigma-Aldrich, Spain; 300 mg/L) and their intestines were extracted, from just after the pyloric caeca to the anus, under sterile conditions. The three intestines were gently stripped with autoclaved tweezers, and the chyme collected from each one was pooled into a 50 mL tube with 10 mL of sterile PBS held on ice. After stripping, the intestines were opened lengthwise and the content from the mucosal layers were insistently but gently scraped with a round edge spatula and pooled into the same 50 mL tubes with the chyme. The remaining intestinal tissue was cut into small pieces of *ca.* 1 cm, placed into 5 mL of sterile PBS in a separate tube, shaken vigorously for 2 min; then the tissue pieces were removed, and the liquid was added to the tube containing the chyme and mucosa collected previously. To homogenize the samples, the content of the tube was transferred to a sterile stomacher bag and triturated at maximum speed for 3 min (Stomacher *Lab-Blender* 400, Seward, United Kingdom) (Fig. 2). An aliquot of 100 µL was spread onto TSA + 0.6% NaCl, which was used as a general medium for the growth of bacteria and fungi, and incubated at 22 °C. Bacterial growth was checked at 24 h. The same protocol was followed with three gilthead seabream that did not receive the mixture of AMs as a control to compare normal bacterial growth in culture media to the AM-treated samples. The process was repeated at 48 h post AM-treatment and growth evaluated after 24 h at 22 °C.

Additionally, to ensure the absence of effects from potential antimicrobial residues in the blood of gilthead seabream, 24 h after the AM gavage three individuals were randomly netted and anesthetized for blood collection via caudal vein with 1 mL heparinized syringes (ref. 303,179, BD Plastik, Canada) and the plasma was separated from the blood by centrifugation (1600×*g*, 10 min, 4 °C). A volume of 100 µL from different plasma dilutions (1:2, 1:10, and 1:20) were placed in equidistant 5 mm diameter-wells made on the surface of Mueller–Hinton media in which a lawn of *E. coli* had previously been spread and the plates were incubated at 22 °C.

A separate group of gilthead seabream (n = 15) were kept fasted during 17 days after the AM administration for testing the effect of the AM treatment on the gut bacterial communities and to record the establishment of the microbiota over time, regardless of the influence of the diet. For this purpose, intestinal samples from gilthead seabream pre-AMs and at 24 h, 8 days and 17 days post-AMs that were continuously fasted during all the period, were collected as described above (chyme + mucosal content + liquid after tissue shaking; Fig. 2) and frozen at − 80 °C until DNA extraction (n = 3 for gilthead seabream pre- and 24 h post-AMs; n = 6 for 8 and 17 days post-AMs since fasting produced very little microbial content).

### Intestinal microbiota transplant

For the preparation of the IMT, 30 Altantic salmon (BW = 240.2 ± 19.3 g) were euthanized with an overdose of MS-222 (> 150 mg/L MS-222) and their intestinal content was extracted following the above-described procedure (Fig. 2), but in this case each sample was resuspended in 10 mL of 0.9% PBS and 10% glycerol, rather than only in PBS, following the recommendations of Quaranta et al.^24^ for handling practices for microbial transplants. Three randomly chosen samples were stored at − 80 °C for DNA extraction, and the rest of the samples were pooled in groups of five and homogenized together in a stomacher (*Lab-Blender* 400), then mixed all together in a sterile 1 L-bottle by constant shaking at 4 °C. This suspension was filtered through sterile gauze into another bottle for reducing the amount of residual partially digested food that might clog the cannula during administration to the recipient fish. The filtered homogenate was immediately frozen at − 80 °C until IMT.

A total of 50 gilthead seabream (BW = 570.5 ± 58.3 g) were anesthetized with 100 mg/L MS-222 and the mixture of AMs was orally and anally administered as described in Fig. 1. Then, fish were randomly separated into two different 2000-L tanks (n = 25), as each tank would be assigned to a diet (salmon and GSB diets). After 24 h, all the specimens from each tank were netted, anesthetized and the thawed bacterial suspension collected from salmon was brought to ambient temperature and administered via oral and anal gavages (1 mL each route) using a cannula as described above. After the IMT, all gilthead sea breams were returned to their respective tanks. No mortality derived from fish handling and IMT was observed.

### Assessment of the diet impact in the establishment of the bacterial communities after the intestinal microbiota transplant

**Figure 3:**
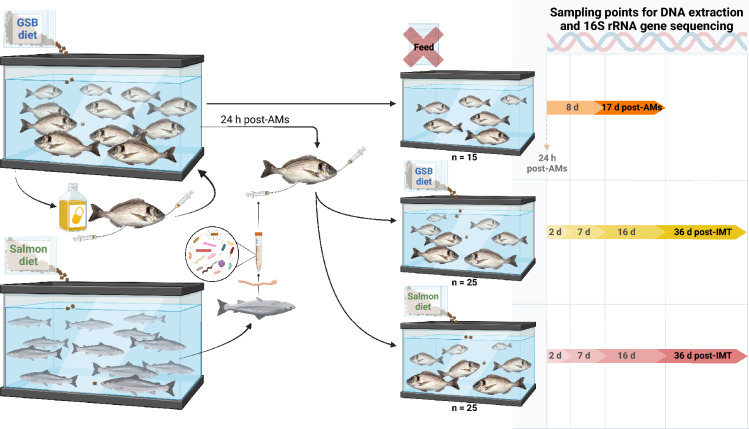
Schematic representation of the intestinal microbiota transplant (IMT) from Atlantic salmon (*Salmo salar*) to gilthead seabream (*Sparus aurata*) and subsequent nutritional assay carried out for assessing the effect of the diet in the gut bacterial communities. After the antimicrobial (AM) treatment, one group of gilthead seabream (n = 15) was fasted for 17 days in order to assess the effect of AMs in the absence of dietary influences. The rest of the gilthead seabream (n = 50) were submitted to the IMT 24 h post-AMs, and in order to assess the effect of the diet, gilthead seabream given the IMT were divided in two tanks (n = 25 per tank): one which continued with the GSB diet, and the other fed with the salmon diet. Created with BioRender.com.

To assess the effect of the diet in the establishment and/or maintenance of bacterial communities after the IMT, gilthead seabream from the first tank continued being fed with the GSB diet, while those from the second tank received the salmon experimental diet. Fish in both tanks were fed by hand three times per day at a feed ratio of 1.5% of the stocked biomass for 36 days. Samples from intestinal content were obtained at different times to study the progression of development over time of the diversity, structure, and composition of the bacterial communities. Sample collection was performed at 2, 7, 16 and 36 days post-IMT (n = 6 per dietary treatment at each time, n = 7 at final time; Fig. 3). At each sampling point after the 2 days, fish BW was measured. Three samples of each diet (salmon and GSB) were also analyzed to compare the microbiota present in them with that from the gut samples.

### DNA extraction and 16S rRNA gene amplification for sequencing

Extractions of DNA were performed following the manufacturer instructions with the DNeasy PowerSoil Pro Kit (ref. 47,016, QIAGEN, Germany). The concentrations and purity of extracted DNA were evaluated by means of a Nanodrop-2000® spectrophotometer (Thermo Fisher Scientific, USA). The DNA concentrations were higher than 20 ng/µL and the A_260_/A_280_ absorbance ratios were higher than 1.80.

The V3-V4 region of the 16S rRNA gene was amplified with the primers 341F (5′-CCTACGGGNGGCWGCAG-3′) and 805R (5′-GACTACHVGGGTATCTAATCC-3′) according to the 16S Metagenomic Sequencing Library Preparation guide^25^. First PCR was performed with Q5® High-Fidelity DNA Polymerase (ref. M0491L, New England BioLabs, USA) using the following programme: a step of 30 s at 98 °C for initial denaturation and polymerase activation, followed by 30 cycles of 10 s at 98 °C, 30 s at 55 °C, 30 s at 72 °C, and 2 min at 72 °C of final extension. After that, a second amplification of 8 cycles was run in order to add the specific barcodes to the templates. The amplified region was then sequenced on an Illumina MiSeq Platform (2 × 300 bp paired-end).

### Data analyses and statistics

Forward and reverse primers were removed from the *fastq* files by means of the Cutadapt tool in the open-source software QIIME2 (v2022.2)^26^. A workflow based on the R package DADA2 (v1.24.0), which is applied to model and correct Illumina-sequenced amplicon errors^27^, was carried out. In brief, reads were subjected to quality filtering, excluding those with a Phred quality score < 28; then, paired-end reads were merged and the sequences with an overlap length < 12 nucleotides, more than 0 mismatches, or identified as chimeras were also removed. During this process, six samples were discarded from the analysis (one from the group gilthead seabream 8 days post-AMs, two from the group 17 days post-AMs, one from the group fed the GSB diet 2 days post-IMT, and two from the group fed the salmon diet 2 days post-IMT). DADA2 resolves differences at the single-nucleotide level and the end products are amplicon sequence variant (ASVs).

Bacterial taxonomy was assigned using the SILVA database (v138.1) as a reference library. A bootstrapping confidence of 80% was established as a cut-off to be considered as a reliable assignment^28^; otherwise, ASVs were classified as unassigned. Template rarefaction curves were obtained using the R package vegan (v2.6-4). Then, all samples were rarefied to the lowest sample depth by subsampling 15,993 reads per sample, which was a representative sample size of the different ASVs occurring in the samples (Supplementary Fig. S1), and normalized by total sum scaling^29^ to calculate the alpha diversity indices of ACE, Shannon, and Faith’s phylogenetic diversity^30,31^. Significant differences in these indices among groups were calculated using the Kruskal–Wallis test followed by the Wilcoxon *post-hoc* test (*P* ≤ 0.05). With the values of alpha diversity obtained from gilthead seabream fed their initial diet and the salmon diet, a linear mixed model was first performed taking the variables diet and time as fixed factors and the sampling order as a random factor, for testing the significant effect of these variables and their interaction, by means of the lme4 package^32^. The association between alpha diversity values and time was analysed with Spearman’s correlation coefficient (*r*_*s*_; *P* ≤ 0.05). For beta diversity and relative abundance, data was normalized by cumulative sum scaling (CSS) which scales counts by dividing the sum of each sample’s counts up to and including a percentile quantile in order to avoid the bias that may be introduced by preferential amplification or sequencing of specific sequences^33^. In this study, we scaled the counts by the 50^th^ percentile of the number of ASVs in each sample for normalization by means of the package metagenomeSeq (v1.38.0)^34^. Beta diversity was approached with the quantitative metrics of Bray–Curtis distance^35^ and weighted UniFrac distance, which was used to estimate similarities among samples based on the phylogenetic relationships of their ASVs^36^. The phylogenetic distances among ASVs used to calculate the indices of Faith’s phylogenetic diversity and weighted UniFrac were also obtained with QIIME2^26^. Results of beta diversity were represented by means of a Principal Coordinate Analysis (PCoA), and a permutational multivariate analyses of variance (PERMANOVA) was used to check significant differences in beta diversity, performing pairwise PERMANOVAs among groups as a *post-hoc* test (*P* ≤ 0.05). The significant effect of the variables diet and time, and their interaction were also tested using a PERMANOVA including both fixed variables. Data on relative abundance among ASVs was analysed by Kruskal–Wallis followed by Wilcoxon test, with *P* set to 0.1 for determination of significance due to the low number of methodological replicates of the diets and of the baseline samples of Atlantic salmon and gilthead seabream. The above-mentioned statistics were partly obtained with the R package microeco (v0.12.0)^37^, which was used to generate figures together with ggplot2 (v3.4.1). Significant differences in fish BW among experimental groups post-IMT were checked by means of a one-way ANOVA with Tukey’s range *post-hoc* test for multiple comparison among groups (*P* ≤ 0.05), after data verification of normal distribution (Shapiro–Wilk test) and homoscedasticity (Levene’s test).

### Ethics declaration

All procedures involving animal care, manipulation and sampling were carried out by trained competent personnel, complied with the Spanish (law 32/2007 and Royal Decree 1201/2015) and the European legislation (EU2010/63). The experimental protocol was authorized by the Ethical Committee of the Institute of Agrifood Research and Technology and the Generalitat of Catalunya (CEEA 11264/2021).

### ARRIVE guidelines

The study was conducted in compliance with the ARRIVE guidelines and all methods were conducted in accordance with relevant guidelines and regulations.

## Results

### Culture-bacterial growth after antimicrobial treatment for establishment of baseline criteria

The preliminary trial to evaluate the efficacy of the AM cocktail and the timing of recovery of the basal microbial community indicated that gut microbiota recovery was rapidly advancing after 48 h, which indicated that the actual IMT trial might be initiated 24 h post-AM treatment. After 24 h of incubation in TSA + 0.6% NaCl media, only 6 CFUs were growing from the intestinal microbiota obtained from the AM-treated fish at 24 h, and the number of CFUs increased to 10^2^ CFUs at 48 h after AM gavage, while the bacterial growth from control animals remained at 10^3^ CFUs in both times. In addition, the absence of clearance zones of growth in the Mueller–Hinton media after 24 h- and 48 h-incubations of the blood of gilthead seabream 24 h post-AM gavage, indicated that there was no risk of retention of antimicrobial residues after 24 h.

### Fish body weight

As expected, fasted gilthead seabream showed lower BW values when compared to the fed groups at all sampling times (Table 1), displaying significant differences with respect to GSB fed the salmon diet at 7 days post-IMT, and with respect to gilthead seabream fed both diets (GSB diet and salmon diet) at 16 days post-IMT (*P* < 0.05). There were no differences seen regarding the administered diet after the IMT (*P* > 0.05).

**Table 1:** Body weight (BW, g) of gilthead seabream (*Sparus aurata*) (GSB) under the different experimental conditions.

	Time after intestinal microbiota transplant		
	7 days	16 days	36 days
Fasted GSB*	524.2 ± 41.7^a^	536.8 ± 49.0^a^	–
GSB fed GSB diet	608.7 ± 106.8^ab^	668.7 ± 59.9^b^	752.4 ± 72.8
GSB fed salmon diet	688.0 ± 73.9^b^	740.3 ± 84.6^b^	769.4 ± 120.8

The days in the table header indicate the number of days after the intestinal microbiota transplant (IMT) from donor Atlantic salmon.

Values are represented as mean ± SD (n = 6 fish individuals per group; n = 7 at 36 days). Significant differences among the three experimental groups at days 7 and 16 are indicated in the same row by the different superscript letters (one-way ANOVA, with Tukey’s range post-hoc test; *P* ≤ 0.05). There were no differences regarding diet after the IMT (GSB fed GSB diet vs. GSB fed salmon diet; Student’s t-test; *P* > 0.05).

*Fasted GSB were not subjected to the IMT and were used as a control to assess the effect of AMs over time and to observe what microbiota profile was recovered in the absence of the influence of feed: in that case, the times 7 and 16 days (post-IMT) correspond to 8 and 17 days post-AMs respectively. The two other groups of fish were submitted to the IMT after which they were fed either the GSB diet or the salmon diet.

### Effect of the antimicrobial treatment on the diversity, structure, and composition of the gut bacterial communities

**Figure 4:**
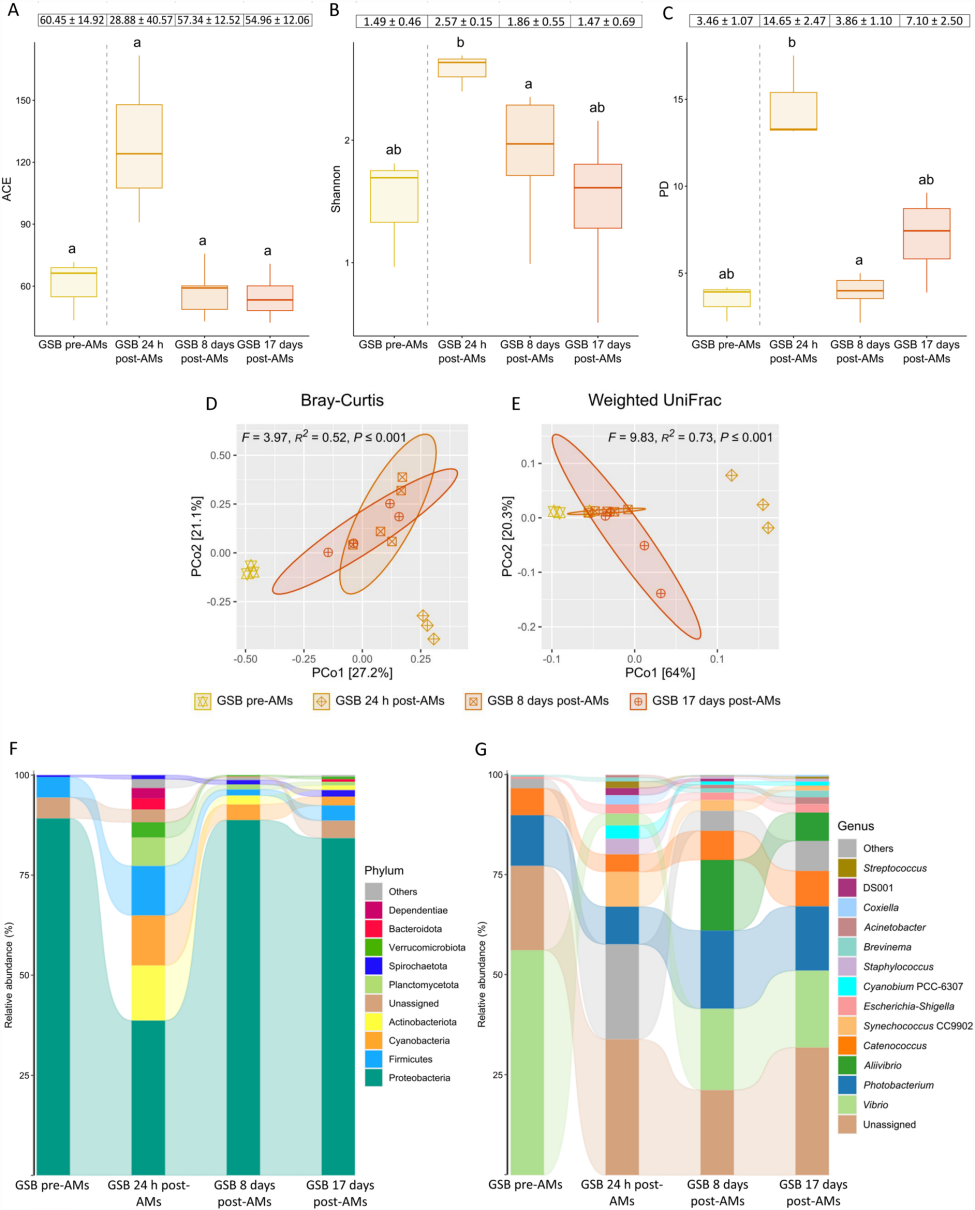
Gut bacterial communities in fasted gilthead seabream (*Sparus aurata*) from the pre- antimicrobial treatment (GSB pre-AMs) during the first 17 days: Alpha diversity measured by (**A**) ACE index, (**B**) Shannon index, and (**C**) Faith’s phylogenetic diversity index (PD). The letters show significant differences among experimental groups (Kruskal–Wallis with Wilcoxon *post-hoc* test; *P* ≤ 0.05). Microbial structure analyzed with (**D**) Bray–Curtis and (**E**) weighted UniFrac distances. Significant differences among experimental groups are shown in black captions (PERMANOVA; *P* ≤ 0.05). Microbial composition at the level of (**F**) phylum and (**G**) genus. Taxa with an abundance < 0.5% are classified as “Others”. Mean ± SD values and significant differences among experimental groups (Kruskal–Wallis with Wilcoxon *post-hoc* test; *P* ≤ 0.1) at the level of phylum and genus can be found at Supplementary Tables S1 and S2, respectively.

Regarding microbial diversity, no differences in the ACE index were observed after the AM treatment in fasted gilthead seabream (Kruskal–Wallis, *P* = 0.067; Fig. 4A). However, when performing the Wilcoxon test between the experimental groups, gilthead seabream at 24 h post-AMs showed a tendency towards increasing estimated richness values with respect to gilthead seabream from the pre-AM treatment (*P* = 0.1), whereas at 8 and 17 days post-AMs there was a downward tendency with respect to specimens from the 24 h post-AMs sampling point (*P* = 0.036; *P* = 0.057, respectively). When using both the Shannon and Faith’s phylogenetic diversity (PD) indices, a significant decrease was observed at 8 days post-AMs with respect to 24 h post-AMs (*P* = 0.036 in both cases; Fig. 4B,C). Likewise, there was a tendency to an increase in diversity at 24 h post-AMs with respect to gilthead seabream pre-AMs (*P* = 0.1), while at 17 days post-AMs tended to decrease with respect to the 24 h post-AMs sampling point (*P* = 0.057 in both cases). Further, the values for the Shannon index were negatively correlated to the number of days that had passed after AM treatment (Spearman’s correlation coefficient *r*_*s*_ = − 0.77, *P* = 0.003).

In terms of beta diversity, when using the Bray–Curtis metric (Fig. 4D), the PCoA distribution of the samples clearly showed that the structure of the bacterial communities was very different among gilthead seabream from the pre-AMs group, and those collected at 24 h post-AMs, and with respect to the other groups post-AMs (*P* < 0.05). In contrast, the distribution of gilthead seabream at 8 days post-AMs and at 17 days post-AMs was very similar (*P* = 0.281). When also considering the phylogenetic relationships among ASVs within samples (Weighted UniFrac; Fig. 4E), the structures of the bacterial communities in gilthead seabream at 17 days post-AMs and from the pre-AMs group resemble each other (*P* = 0.060) more than the groups at 8 days post-AMs and at 17 days post-AMs (*P* = 0.031). In that latter case, except between gilthead seabream from the pre-AMs group and at 17 days post-AMs, the remaining were different among them (*P* < 0.05).

Regarding composition of gut bacterial communities, the relative abundance of the phylum Proteobacteria was reduced by 50% at 24 h after the AM treatment (*P* ≤ 0.1; Fig. 4F; Supplementary Table S1). On the other hand, the relative abundance of the rest of phyla ≥ 0.5% increased (*P* ≤ 0.1), except for Spirochaetota (*P* > 0.1). At eight days post-AMs, the relative abundances of Proteobacteria, Planctomycetota, Verrucomicrobiota, Bacteroidota and Dependientae were already re-established with respect to gilthead seabream from the pre-AMs group (*P* > 0.1) and they were maintained in the same range at 17 days post-AMs. The relative abundance of the other phyla (Firmicutes and Actinobacteriota) did not reach values similar to the initial ones until 17 days post-AMs, except for Cyanobacteria, that showed a gradual decrease after the AM treatment that was maintained at 17 days (*P* ≤ 0.1). At the level of genus, the AM treatment induced a change in the relative abundance of 70% of the represented genera (those with a relative abundance ≥ 0.5% excluding those classified as unassigned) at 24 h post-AMs; in particular, a decrease in *Vibrio*, while an increase in *Synechococcus* CC9902, *Escherichia-Shigella*, *Cyanobium* PCC-6307, *Staphylococcus*, *Acinetobacter*, *Coxiella*, DS001, and *Streptococcus* was observed (*P* ≤ 0.1; Fig. 4G; Supplementary Table S2). *Photobacterium*, *Catenococcus* and *Brevinema* were the only genera to remain unchanged at 24 h post-AMs. At 8 days post-AMs, the relative abundances of 54% of the genera that were originally represented in the baseline samples were already restored, including *Cyanobium* PCC-6307, *Staphylococcus*, *Coxiella*, DS001, and *Streptococcus*. The appearance of *Aliivibrio* at this sampling time was remarkable, as this was not present in gilthead seabream at 24 h post-AMs and pre-AMs (*P* ≤ 0.1). At 17 days post-AMs, the relative abundance of *Synechococcus* CC9902 and *Acinetobacter* was also re-established with respect to gilthead seabream from the pre-AMs group, so that 85% of the genera contained a relative abundance similar to the baseline microbiota, and only that of *Vibrio* and *Escherichia-Shigella* was different at the final sampling point of day 17 (*P* ≤ 0.1).

### Effect of the intestinal microbiota transplant and species-specific diets on the diversity, structure and composition of the gut bacterial communities

There was no significant effect of the time (*F* = 0.83, *P* = 0.516) and the diet (*F* = 0.15, *P* = 0.704) on the ACE index, but there was for their interaction (*F* = 11.48, *P* ≤ 0.001). When using the Shannon and Faith’s phylogenetic diversity (PD) indices, there was significance on both variables; time (Shannon: *F* = 4.40, *P* = 0.005; PD: *F* = 9.85, *P* ≤ 0.001) and diet (Shannon: *F* = 5.46, *P* = 0.025; PD: *F* = 18.17, *P* ≤ 0.001), as well as on their interaction (Shannon: *F* = 3.21, *P* = 0.023; PD: *F* = 7.36, *P* ≤ 0.001). Thus, the significant differences among experimental groups regarding these two variables were individually evaluated.

**Figure 5:**
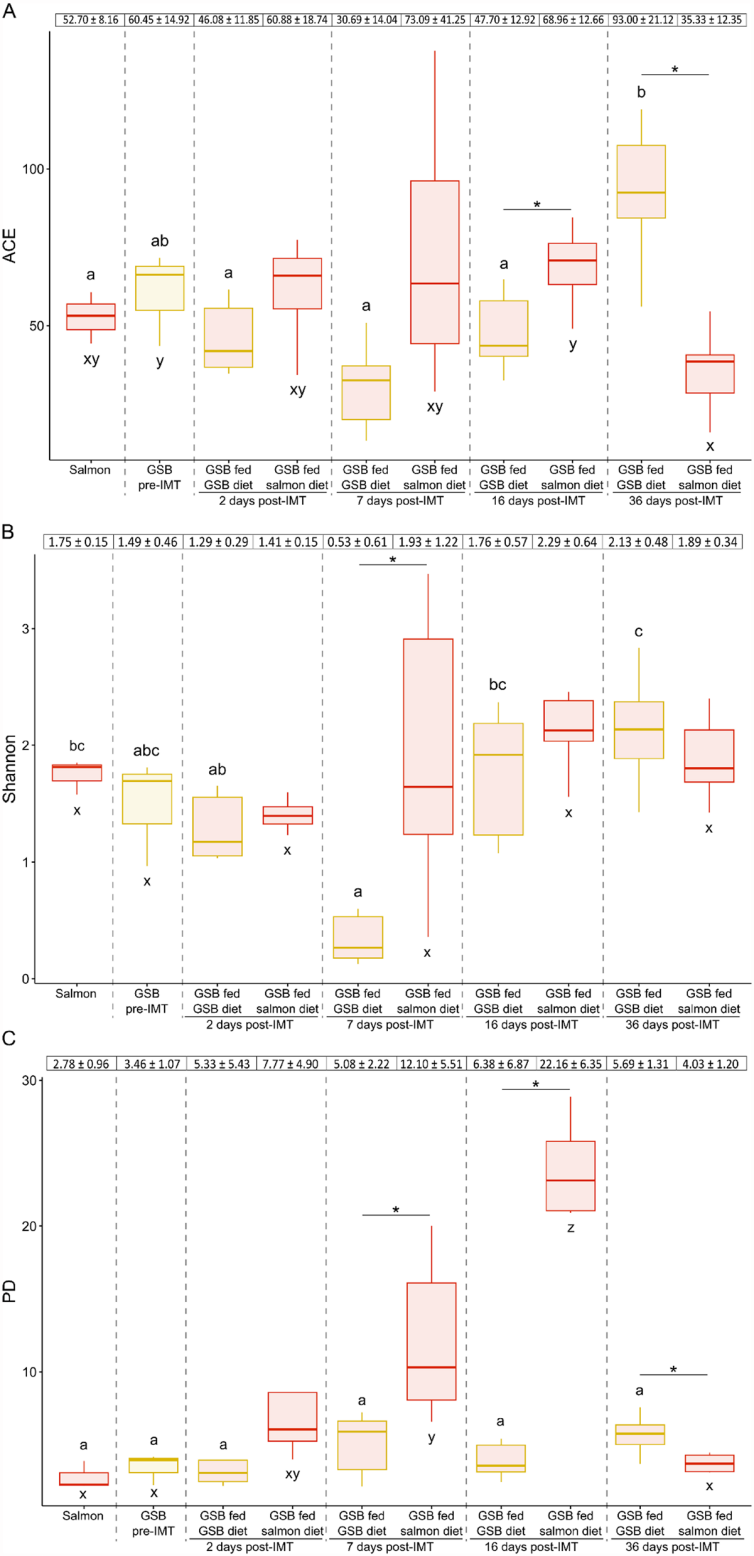
Microbial alpha diversity of the gut bacterial communities in Atlantic salmon (*Salmo salar*), in gilthead seabream (*Sparus aurata*) previous to the intestinal microbiota transplant from donor Atlantic salmon specimens (GSB pre-IMT and after AM treatment) and in gilthead seabream fed either their typical diet (GSB fed GSB diet) or the Atlantic salmon diet (GSB fed salmon diet) at 2, 7, 16 and 36 days post-IMT: (**A**) ACE index, (**B**) Shannon index, and (**C**) Faith’s phylogenetic diversity index (PD). The top table represents the index values per group as mean ± SD. Box plots represents the minimum, maximum and the median of the sample values obtained from the diversity indices, and the letters show significant differences among experimental groups (Kruskal–Wallis with Wilcoxon *post-hoc* test; *P* ≤ 0.05).

For the ACE index, gilthead seabream fed the GSB diet after the IMT and until day 16 post-IMT (included) displayed a similar diversity than that from the pre-IMT samples from Atlantic salmon and gilthead seabream (Fig. 5A). However, at 36 days post-IMT, gilthead seabream fed the GSB diet showed a microbial diversity different to the one of Atlantic salmon (*P* < 0.05), although still similar to the pre-IMT group (*P* > 0.05). Completely the opposite was seen for gilthead seabream fed the salmon diet, which did not display significant differences over time with respect to the Atlantic salmon microbiota (*P* > 0.05); however, at 36 days post-IMT, microbial diversity was lower than in gilthead seabream from the pre-IMT (*P* < 0.05). Values of the ACE index in gilthead seabream fed the GSB diet were positively correlated with the time of post-IMT from 7 days onwards (Spearman’s correlation coefficient *r*_*s*_ = 0.85, *P* ≤ 0.001). Differences regarding the species-specific diet were observed at 16 and 36 days post-IMT (*P* < 0.05). Similarly, no differences in the Shannon index for gilthead seabream fed the GSB diet were found at any time post-IMT with respect to the pre-IMT group (*P* > 0.05); while at 7 days there was a significant decrease in comparison to the Atlantic salmon microbial diversity (*P* < 0.05), but afterwards it increased again reaching similar values to those seen for Atlantic salmon (Fig. 5B). Indeed, these values of Shannon’s alpha diversity found in gilthead seabream fed the GSB diet were also positively correlated with the time of post-IMT from 7 days onwards (*r*_*s*_ = 0.69, *P* ≤ 0.001). On the other hand, fish fed with the salmon diet after the IMT did not show differences among sampling times nor with the baseline microbial diversities from gilthead seabream or donor Atlantic salmon specimens (*P* > 0.05). In this case, significant differences regarding each species-specific diet were only observed at 7 days post-IMT (*P* < 0.05). Faith’s phylogenetic diversity in gilthead seabream fed the GSB diet did not vary among times and with respect to the values from the baseline fishes (Atlantic salmon microbiota and gilthead seabream from the pre-IMT group) (Fig. 5C). In contrast, gilthead seabream fed the salmon diet showed higher PD values at 7 and 16 days post-IMT than the Atlantic salmon microbiota and gilthead seabream from the pre-IMT group (*P* < 0.05). In fact, there was a positive correlation between the samples from days post-IMT and PD from 2 days post-IMT to 16 days post-IMT groups (*r*_*s*_ = 0.79, *P* ≤ 0.001); whereas at 36 days the alpha diversity values were restored with respect to the initial ones of Atlantic salmon and gilthead seabream from pre-IMT groups (*P* > 0.05). Furthermore, there were significant differences in PD values between gilthead seabream fed the GSB diet and those fed the salmon diet from 7 days onwards (*P* < 0.05).

In terms of beta diversity, after the IMT the gut microbial structure of gilthead seabream was significantly affected by both factors: the diet (Bray–Curtis: *F* = 4.71, *R*^*2*^ = 0.07, *P* ≤ 0.001; weighted UniFrac: *F* = 17.82, *R*^*2*^ = 0.17, *P* ≤ 0.001) and the time after the IMT (Bray–Curtis: *F* = 4.81, *R*^*2*^ = 0.22, *P* ≤ 0.001; weighted UniFrac: *F* = 10.33, *R*^*2*^ = 0.29, *P* ≤ 0.001); as well as by their interaction (Bray–Curtis: *F* = 2.39, *R*^*2*^ = 0.11, *P* ≤ 0.001; weighted UniFrac: *F* = 6.28, *R*^*2*^ = 0.18, *P* ≤ 0.001). Therefore, in order to find out the individual effect of the diet on the structure of the gut bacterial communities post-IMT, the different distribution in the PCoA and significant differences among gilthead seabream fed both types of diets, as well as among the diets themselves, were separately analysed at the different sampling points.

**Figure 6:**
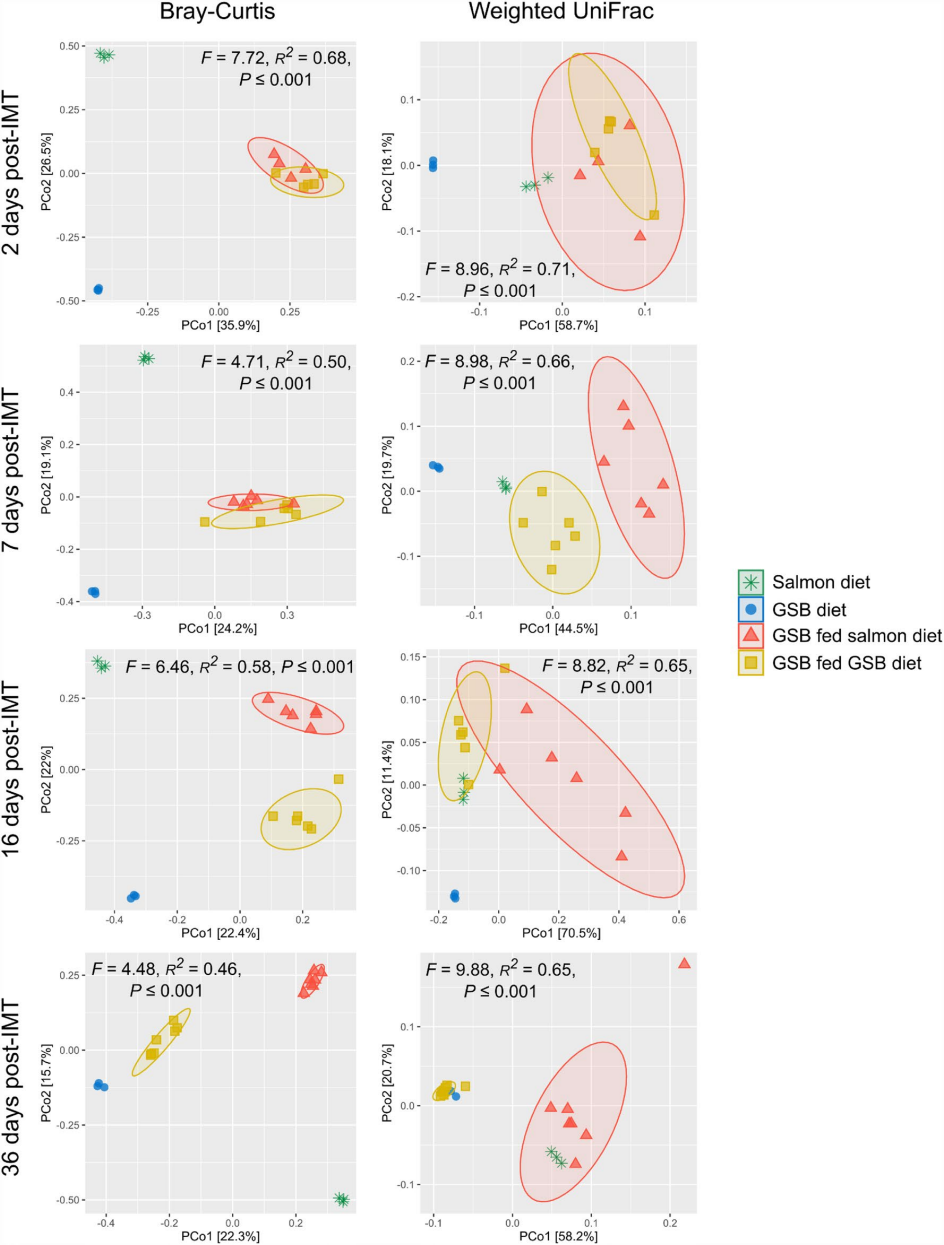
Microbial structure of the gut bacterial communities in gilthead seabream (*Sparus aurata*) fed either their typical diet (GSB fed GSB diet) or the Atlantic salmon diet (GSB fed salmon diet) at 2, 7, 16 and 36 days after the intestinal microbiota transplant from donor Atlantic salmon (post-IMT), as well as of GSB and salmon diets. Beta diversity was analyzed with the metrics of Bray–Curtis and weighted UniFrac distances, and represented by individual distributions in the principal coordinate analyses. Significant differences among experimental groups are shown (PERMANOVA; *P* ≤ 0.05).

Pairwise differences were observed among the four experimental groups at 2 days post-IMT when using the Bray–Curtis metric (*P* < 0.05) except for the salmon diet and the GSB diet, with *P* = 0.1 in all times, which may be attributed to the low number of replicates (n = 3 for both diets). While the diets appeared separately in the PCoA between them and with respect to gilthead seabream irrespective of the administered diet, fish fed each diet were grouped close to each other (Fig. 6; despite *F* = 1.75, *R*^*2*^ = 0.20, *P* = 0.035). Pairwise significant differences among all groups were maintained at the different sampling times (*P* < 0.05), whereas the groups fed with the salmon diet and the GSB diet were found further apart as the days passed away. After 36 days from the IMT, the microbial structure of gilthead seabream fed the GSB diet was closer to the GSB diet (*F* = 3.08, *R*^*2*^ = 0.28, *P* = 0.014) than to their congeners fed the salmon diet (*F* = 3.76, *R*^*2*^ = 0.24, *P* = 0.003). When approaching the beta diversity with the weighted UniFrac metric, all fish displayed a similar microbial structure regardless of the diet administered after the IMT (*F* = 0.69, *R*^*2*^ = 0.09, *P* = 0.572; Fig. 6). During the rest of times, all the groups remained separate (*P* < 0.05), with the exception of the salmon diet and gilthead seabream fed the salmon diet at 36 days post-IMT, which clustered together (*F* = 2.33, *R*^*2*^ = 0.23, *P* = 0.083). Although the distances of weighted UniFrac in the GSB diet and in gilthead seabream fed the GSB diet 36 days post-IMT were significantly different (*F* = 9.43, *R*^*2*^ = 0.54, *P* = 0.012), they showed a very close distribution in the PCoA.

**Figure 7:**
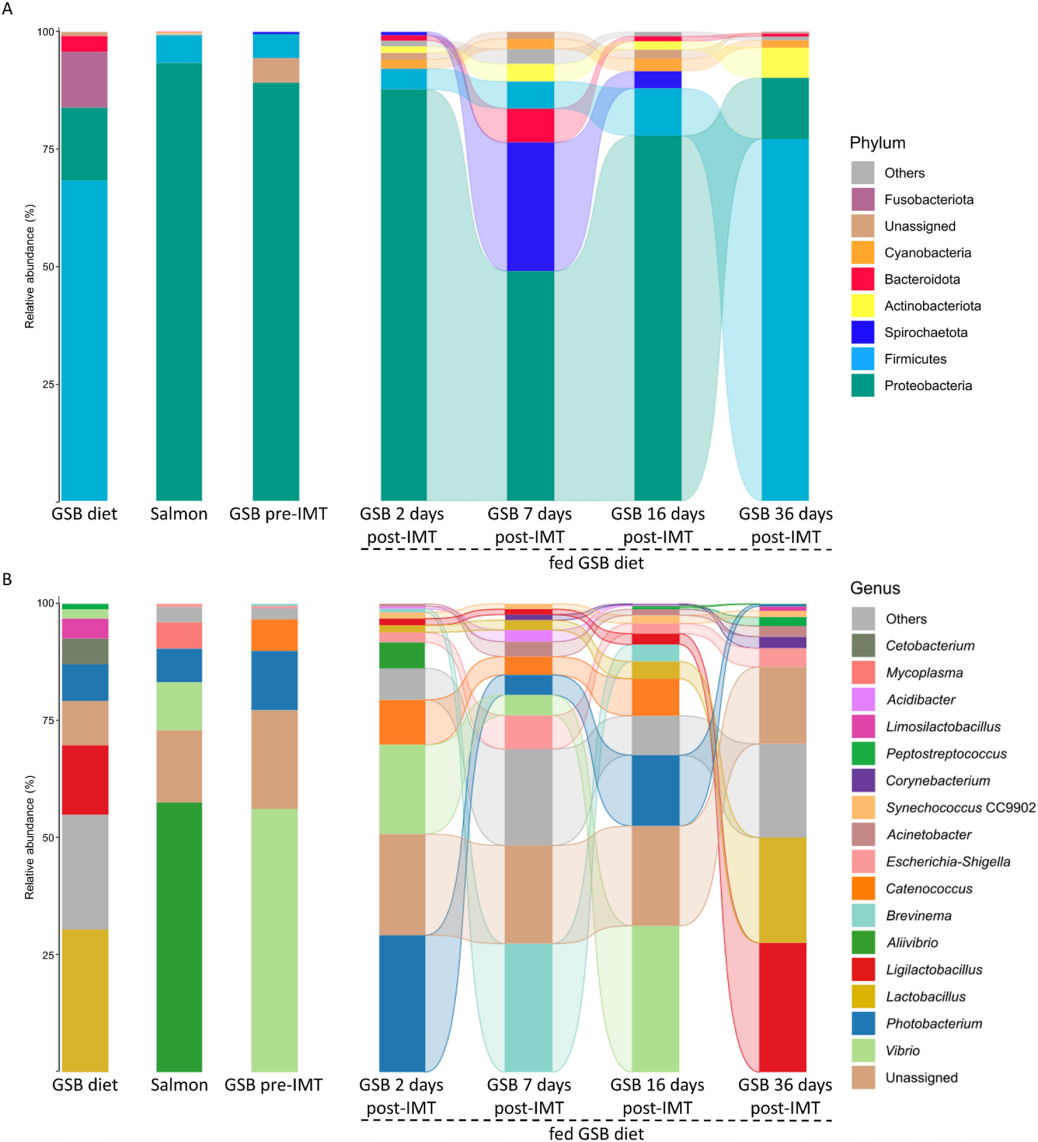
Contrasting control treatments with gilthead seabream (*Sparus aurata*) fed an experimental gilthead seabream diet (GSB diet): Mean relative abundances of the gut bacterial communities in GSB diet, Atlantic salmon (microbiota donor), gilthead seabream previous to the intestinal microbiota transplant (GSB pre-IMT and after AM treatment) and in gilthead seabream fed the GSB diet at 2, 7, 16 and 36 days post-IMT (**A**) at the level of phylum, and (**B**) at the level of genus. Taxa with an abundance < 0.5% are classified as “Others”. Mean ± SD values for phyla and genera are compiled in Supplementary Tables S3 and S4, respectively. Significant differences among experimental groups (Kruskal–Wallis with Wilcoxon *post-hoc* test; *P* ≤ 0.1) at the level of phylum and genus can be found in Supplementary Figs. S2 and S3, respectively.

Regarding microbial composition, we first compared the different relative abundances of phyla and genera in gilthead seabream from the post-IMT groups, which were fed throughout the trial with the GSB diet, to the gilthead seabream from the pre-IMT group, the Atlantic salmon microbiota, and the administered diet (GSB diet). Thus, at two days post-IMT, all the phyla ≥ 0.5% had a relative abundance like that of Atlantic salmon microbiota and gilthead seabream from the pre-IMT group (*P* > 0.1; Fig. 7A; Supplementary Table S3 and Supplementary Fig. S2). However, the diet did show differences with respect to the fish in the relative abundance of the following phyla: for Proteobacteria it was higher in gilthead seabream 2 days post-IMT than in the GSB diet, while for Firmicutes and Fusobacteriota it was lower (*P* ≤ 0.1), the latter remaining at 0% from this time onwards. These differences were maintained at days 7 and 16, with an increase in Spirochaetota also appearing with respect to the GSB diet and Atlantic salmon microbiota. At 7 days post-IMT, a decrease in the relative abundance of Proteobacteria with respect to the baseline fishes (Atlantic salmon microbiota and gilthead seabream from the pre-IMT group) was also observed (*P* ≤ 0.1). At the final sampling point (36 days post-IMT), the relative abundance for Proteobacteria was similar to that of the GSB diet, for Spirochaetota it was similar to the GSB diet and to the microbiota of Atlantic salmon (0%), and for Fusobacteriota it resembled that of Atlantic salmon microbiota and gilthead seabream from the pre-IMT groups (0%). In contrast, the relative abundance for Bacteroidota and Cyanobacteria was similar with respect to the baseline samples from fishes (Atlantic salmon microbiota and gilthead seabream from the pre-IMT group) and GSB diet (very close to 0%; *P* > 0.1). The relative abundance of Firmicutes at the final sampling point was very close to that of the GSB diet (gilthead seabream 36 days post-IMT: 77.2 ± 4.6%; GSB diet: 68.3 ± 6.9%) despite *P* < 0.1 (*P* = 0.063).

At the level of genus, the relative abundance of *Acinetobacter*, *Synechococcus* CC9902, *Corynebacterium*, *Peptostreptococcus*, and *Acidibacter* in gilthead seabream fed the GSB diet from 2 days until 16 days post-IMT (included) were in the same range as for the GSB diet, Atlantic salmon microbiota and gilthead seabream from the pre-IMT group (*P* > 0.1; Fig. 7B; Supplementary Table S4 and Supplementary Fig. S3). Noteworthy was the increase in relative abundance of *Brevinema* at 7 days post-IMT, a genus common to gilthead seabream from the pre-IMT group, though this was reduced again at 16 days post-IMT (*P* ≤ 0.1). Additionally, *Catenococcus*, a genus exclusive from gilthead seabream from the pre-IMT group (which was not present in Atlantic salmon microbiota nor in GSB diet), was maintained at a similar relative abundance to the gilthead seabream from the pre-IMT group during 2, 7 and 16 days post-IMT, but it was not observed at 36 days (0%). The dynamics of *Aliivibrio* and *Mycoplasma* were also remarkable, because neither were found in the gilthead seabream from the pre-IMT group nor in the GSB diet, but they were present in the Atlantic salmon microbiota. In particular, *Aliivibrio* appeared at 2 days-post IMT in gilthead seabream, but its presence was not sustained over time, and *Mycoplasma* was not found at any time. On the other hand, *Cetobacterium* appeared in the GSB diet, while its relative abundance was 0% in the rest of the post-IMT fish, as in the baseline fishes (Atlantic salmon microbiota and gilthead seabream from the pre-IMT group). At the final sampling time, only 31% of the represented genera had a similar relative abundance to that from the gilthead seabream from the pre-IMT group, and 31% of the genera also displayed similar relative abundances to those of the Atlantic salmon microbiota. Thus, the effect of the donor and host microbial profiles lost significance over time, considering that at 2 days post-IMT 69% of the genera contained a similar relative abundance to that from the gilthead seabream from the pre-IMT group and 50% of the genera showed similar relative abundances to those of the Atlantic salmon microbiota. On the other hand, the administered diet gained significance at 36 days post-IMT, showing more than 56% of genera with a relative abundance similar to the GSB diet group with respect to an initial similarity of 44% at 2 days post-IMT in gilthead seabream fed the GSB diet. It was worth noting the increase in relative abundance of *Lactobacillus* and *Ligilactobacillus* with respect to the previous sampling point (16 days post-IMT), which were common to the GSB diet and they did not appear in the Atlantic salmon microbiota or gilthead seabream from the pre-IMT group (*P* ≤ 0.1).

**Figure 8:**
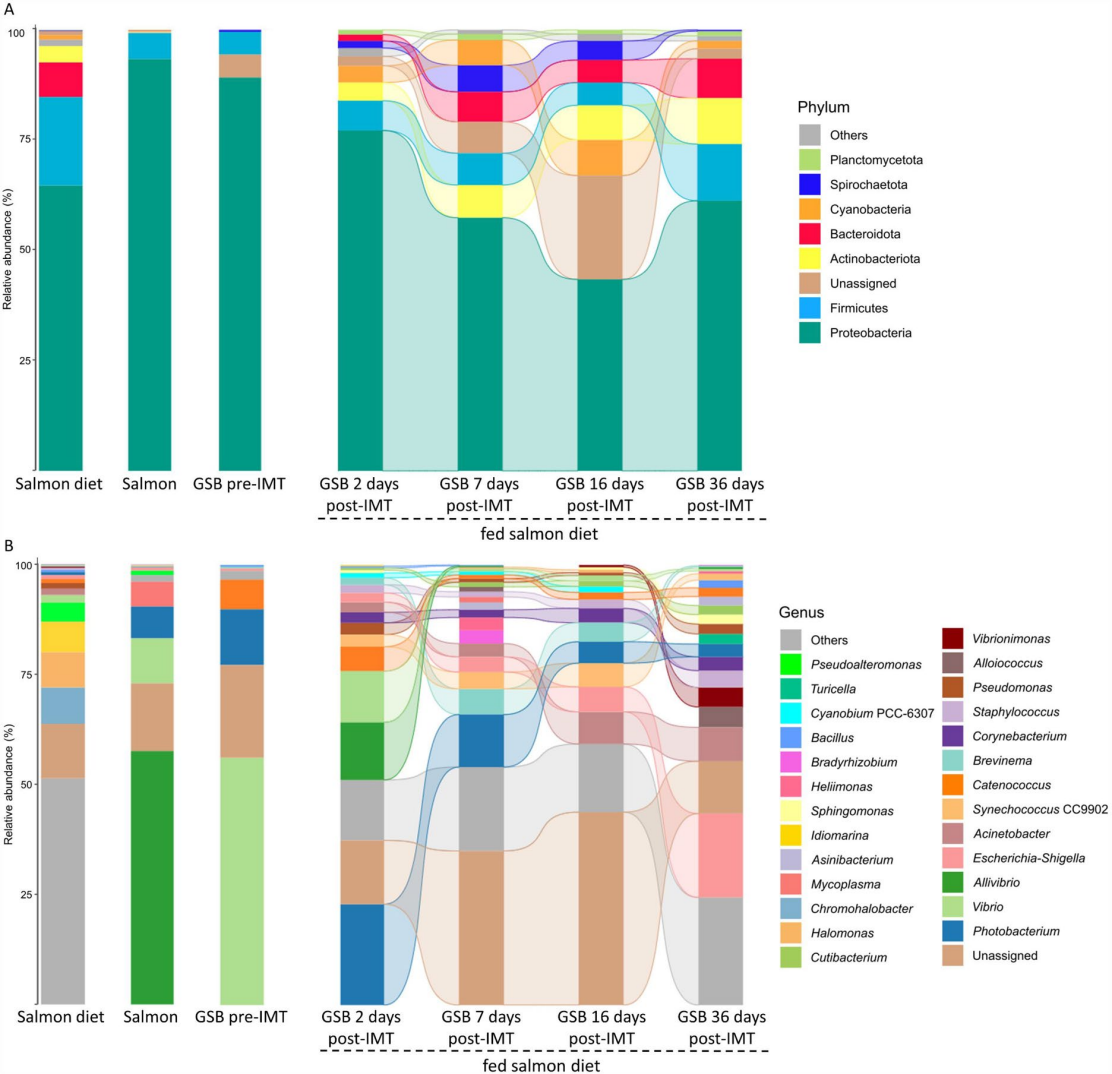
Contrasting control treatments with gilthead seabream (*Sparus aurata*) fed an experimental salmon diet: mean relative abundances of the gut bacterial communities in gilthead seabream fed the salmon diet, Atlantic salmon (microbiota donor), gilthead seabream previous to the intestinal microbiota transplant (GSB pre-IMT and after AM treatment) and in gilthead seabream fed the salmon diet at 2, 7, 16 and 36 days post-IMT (**A**) at the level of phylum, and (**B**) at the level of genus. Taxa with an abundance < 0.5% are classified as “Others”. Mean ± SD values for phyla and genera are compiled at Supplementary Tables S5 and S6, respectively. Significant differences among experimental groups (Kruskal–Wallis with Wilcoxon *post-hoc* test; *P* ≤ 0.1) at the level of phylum and genus can be found in Supplementary Figs. S4 and S5, respectively.

There were distinctly different results when we compared the microbial composition in gilthead seabream from the post-IMT group, which were fed during all the trial with the salmon diet, to the gilthead seabream from the pre-IMT group, the Atlantic salmon microbiota and the administered diet (salmon diet). At 2 days post-IMT, all the phyla displayed a similar relative abundance to that of the donors (*P* > 0.1; Fig. 8A; Supplementary Table S5 and Supplementary Fig. S4). In the same way, the only phylum which showed a differential relative abundance with respect to gilthead seabream from the pre-IMT group was Bacteroidota, which did not appear in any sample of gilthead seabream from the pre-IMT group (*P* ≤ 0.1). The abundance of this phylum ranged between 1.4 and 8.9% over time, not showing significant differences with respect to the salmon diet at any time (with a mean relative abundance of 7.9 ± 1.5%; *P* > 0.1). Only the phylum Firmicutes showed significant differences at all sampling points after the IMT with respect to the salmon diet (20.1 ± 3.0%; these values were always lower in GSB post-IMT: 5.1–12.9%; *P* ≤ 0.1). The relative abundance of this phylum over time was maintained in the same range as for gilthead seabream from the pre-IMT group (5.1 ± 1.1%) and the microbiota of the donors (5.9 ± 2.2%) (*P* > 0.1). On the other side, the relative abundance of Proteobacteria prominently decreased from 7 days post-IMT onwards with respect to donors’ microbiota and gilthead seabream from the pre-IMT group, while that of Actinobacteriota and Bacteroidota increased (*P* ≤ 0.1). Although the relative abundance of Cyanobacteria and Spirochaetota was also higher in gilthead seabream at 7 and 16 days post-IMT with respect to both reference microbiota (Atlantic salmon and gilthead seabream before the AM treatment) (*P* ≤ 0.1), these significant differences were not sustained at 36 days post-IMT (*P* > 0.1). The relative abundance of Spirochaetota at 7 and 16 days post-IMT also displayed significant differences with respect to that of the salmon diet, which was of 0.2 ± 0.3% (*P* ≤ 0.1).

At the level of genus, no significant differences were observed in the relative abundance of *Corynebacterium*, *Staphylococcus*, *Pseudomonas*, *Alloiococcus*, *Cutibacterium*, *Asinibacterium*, *Sphingomonas*, *Heliimonas*, *Bradyrhizobium*, *Cyanobium* PCC-6307 and *Turicella* in gilthead seabream fed the salmon diet at any of the times post-IMT with respect to the microbiota of donors, gilthead seabream from the pre-IMT group nor the salmon diet (*P* > 0.1; Fig. 8B; Supplementary Table S6 and Supplementary Fig. S5). Although immediately after the microbiota transplant (2 days post-IMT), the relative abundances of *Acinetobacter*, *Synechococcus* CC9902, *Brevinema*, *Vibrionimonas*, and *Bacillus* were also within the same range (very close to 0%) as in the two baseline groups (gilthead seabream from the pre-IMT group, and Atlantic salmon microbiota) and in the salmon diet, their abundances tended to increase and changed over time (*P* ≤ 0.1). Conversely, the relative abundance of *Escherichia-Shigella* was significantly higher in all the times post-IMT than in Atlantic salmon microbiota, in gilthead seabream from the pre-IMT group and in the salmon diet, especially at 36 days post-IMT (19.1 ± 10.6%; *P* ≤ 0.1). Additionally, the relative abundances of *Aliivibrio* and *Mycoplasma* tended to decrease after the IMT in comparison to the microbiota of donors (*P* ≤ 0.1). Regarding differences in composition maintained over time with respect to the gilthead seabream from the pre-IMT group, the relative abundance of *Vibrio* decreased around 50% after the IMT (*P* ≤ 0.1). In comparison to the salmon diet, *Halomonas*, *Chromohalobacter*, *Idiomarina*, and *Pseudoalteromonas* showed a lower relative abundance in all sampled times post-IMT. At the end of the study (36 days post-IMT), 77% of the represented genera (≥ 0.5% and excluding those classified as unassigned) showed a relative abundance similar to that for the Atlantic salmon microbiota, the same percentage as when compared to gilthead seabream from the pre-IMT group. In terms of the salmon diet, the relative abundances of 65% represented genera matched with those of gilthead seabream fed the salmon diet at 36 days post-IMT.

### Comparison of the microbial communities at 7 and 16 days post-IMT with respect to the baseline group not submitted to the intestinal microbiota transplant

**Figure 9:**
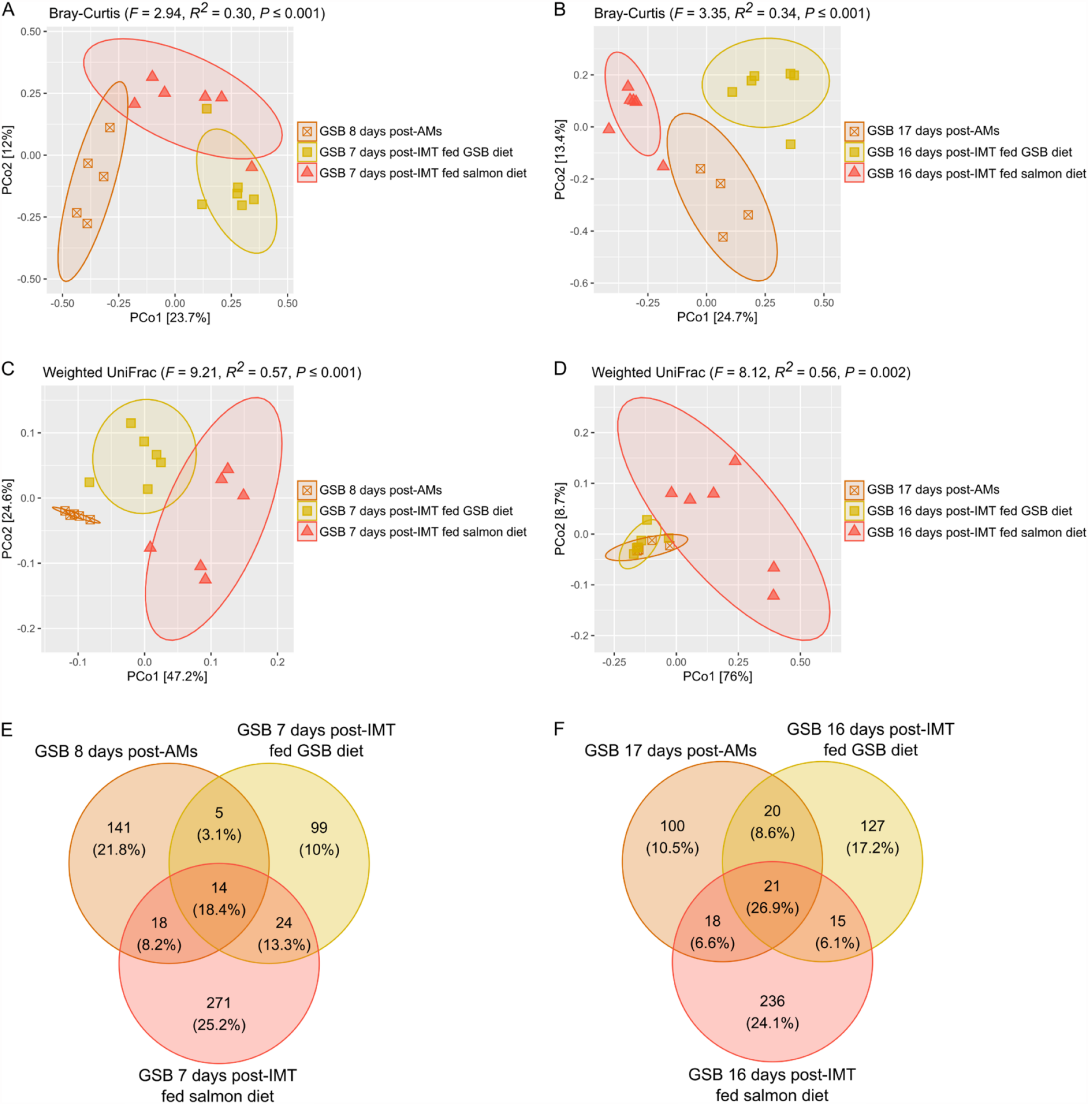
Microbial structure of the gut bacterial communities in gilthead seabream (*Sparus aurata*) from the group which was fasted after the antimicrobial treatment (post-AMs) and the groups which were submitted to the intestinal microbiota transplant and then fed either their typical diet (GSB fed GSB diet) or the Atlantic salmon diet (GSB fed salmon diet). Beta diversity was analyzed using (**A**,**B**) Bray Curtis, and (**C**,**D**) weighted UniFrac distances, at 7 and 16 days post-IMT (8 and 17 days post-AMs), respectively. Venn diagram plotting the number of unique and shared ASVs (and relative abundance %) of the three groups (**E**) at 7 days post-IMT, and (**F**) at 16 days post-IMT.

Beta diversity values were statistically compared among gilthead seabream fed the GSB and the salmon diets and the baseline group not submitted to the IMT (fish fasted after the AM treatment). Significant differences were found among the three groups at both 7 and 16 days post-IMT when Bray–Curtis distances were used (*P* < 0.05; Fig. 9A,B). Considering phylogenetic relations (weighted UniFrac distances), there were also significant differences among the three groups at 7 days (*P* < 0.05; Fig. 9C). However, at 16 days post-IMT, gilthead seabream fed the GSB diet, and the fasted fish not submitted to the IMT were similar (*F* = 1.20, *R*^*2*^ = 0.13, *P* = 0.30; Fig. 9D), while gilthead seabream fed the salmon diet showed significant differences when compared to both groups (*P* < 0.05).

When representing the Venn diagram with the ASVs found in the three groups, a total of 14 and 21 ASVs where shared among the three groups at 7 and 16 days post-IMT, which constituted a total of 18.4% and 26.9% relative abundance, respectively (Fig. 9E,F).

## Discussion

In this study, we have undertaken an experiment to investigate the progression of development of the microbiota when the intestinal microbiota is transplanted into a new host that is then fed with two different diets: the homologous diet given the new host (recipient) and the heterologous diet previously given to the donor Atlantic salmon. To understand what is meant by autochthonous microbiota, we additionally followed the progression of recovery of the native microbiota during 17 days under fasting conditions, after “erasing” most of the microbiota with a cocktail of multiple broad-spectrum antibiotics.

### Antimicrobial treatment effects

Under current experimental conditions, the gut bacterial communities of the baseline gilthead seabream (pre-AMs) were dominated by the phylum Proteobacteria, with *Vibrio* and *Photobacterium* as the most dominant genera, results that were in agreement with previous studies in gilthead seabream^38,39^. In this study, the preliminary trial using a limited number of fish to establish the proper timing of IMT administration after the AM treatment showed that the relative abundance of the phyla Proteobacteria dramatically decreased at 24 h post-AMs, in line with the significant decrease of the genus *Vibrio* and, to a lesser extent, of *Photobacterium* and *Catenococcus*, which also belong to this phylum. However, based on existing trials in humans and other animal species, when administering the AM combination described herein, there could have been expected an increase in the abundance of Proteobacteria^40–43^. Under current experimental conditions, the decrease of Proteobacteria may be caused by the lower concentration of Vancomycin (0.02 g/L) in comparison to the former studies (0.25–0.5 g/L), which usually leads to a relative boost in the abundance of this phylum when supplied individually^44^, likely due to elimination of other competing species, or the possibility of the antibiotic serving as a carbon/energy source for resistant strains/species^45^. The other antibiotics present in the AM cocktail used in this study (Metronidazole, Neomycin, Ampicillin) have been reported to have a more significant effect on the increase of the abundance of other phyla such as Bacteroidota or Actinobacteriota depending on the host species and on the different experimental conditions of each assay^40–43^. On the other hand, and in agreement with the above-mentioned studies, despite the impact of the AM cocktail on the relative abundance of *Vibrio*, *Photobacterium* and *Catenococcus*, other genera belonging to Proteobacteria did show a significant increase, such as *Coxiella*, *Escherichia-Shigella*, and *Acinetobacter*. The changes in abundance of Proteobacteria members were coupled to an increase in the relative abundance of strains belonging to Firmicutes (i.e., bacteria from the genera *Staphylococcus* and *Streptococcus*), and the appearance of new ones that were not present before the AM treatment, such as Cyanobacteria (*Synechococcus* CC9902, *Cyanobium* PCC-6307) and Actinobacteriota (DS001), among others. The increase in the relative abundance of Firmicutes may be caused by the reduced competition for colonizing space and available resources in the intestine because of Proteobacteria death or growth inhibition by the AMs, or just be a bias inherent to the complexity of analyzing microbiota datasets, as they are compositional (that is, each sample consists of proportions of various organisms with a sum constrained to a constant). In this sense, when approaching a 16S rRNA gene experiment with relative abundances, if a taxon goes up while another goes down, it is not possible to know if that occurred just because one taxon has increased, or because the other has decreased, or both^46^. On the other hand, the emergence of taxa which were not present before the AM treatment (members of Actinobacteriota, Cyanobacteria, Planctomycetota, Verrucomicrobiota, Bacteroidota and Dependientae) was reflected in the tendency towards increased richness (ACE), diversity (Shannon) and phylogenetic diversity (PD) values. However, the culture-dependent bacterial growth test showed that most of the gut content lacked viable bacteria after the first 24 h, using TSA + 0.6% NaCl media, which suggested that most of the bacteria sequenced from this sampling time were not viable on the chosen media, while the surviving taxa from the microbiota seemed to readily re-colonize the gut within 48 h after the AM treatment, as evinced by growth on culture media. Nonetheless, it is also important to consider that a large portion of the microbiota is not culturable, or they need more selective media to grow^47^, meaning that the above-mentioned approach was only a proxy of the proportion of viable bacteria culturable on TSA + 0.6% NaCl medium, but we could not determine the total viability of the microbiota. Consequently, we decided to select the time 24 h post-AMs as the best time to perform the IMT from donor Atlantic salmon. Furthermore, among the fasted group, sequencing results showed a gradual trend at 8- and 17-days post-AMs towards re-establishing the microbiota profile that existed before the AM-treatment. At 17 days post-AMs, the only represented genera showing significant differences with respect to the baseline gilthead seabream were *Vibrio* and *Escherichia-Shigella*. The reestablishment of the initial gut microbiota might be facilitated by many biotic and abiotic factors, such as the environmental pressures during the trial, including water temperature and salinity^48,49^, the environmental microbiota composition^50^, the fish origin^51^, species, sex, age^52^, and host’s genetic background^53^. Meanwhile, the few changes observed with respect to the gilthead seabream pre-AM treatment (baseline state) may be caused by possible residual effects of the antibiotics and/or by the effect of the fasting conditions. Regarding the fasting state, for instance, many *Vibrio* species acting as symbionts produce hydrolytic enzymes (i.e., amylase, lipase, cellulase, and chitinase), which contribute to the breakdown of dietary components^54^, so the pronounced decrease in *Vibrio* relative abundance may be a response to the absence of feed in the digestive tract of fish. Whatever the reason, the restoration of the intestinal microbiota composition was in line with the recovery of initial values of alpha diversity over time, which was particularly evident for the Shannon diversity index, which showed a negative correlation with the number of days post-AMs until reaching values like those of the baseline samples of gilthead seabream pre-AMs after 17 days (*r*_*s*_ = − 0.77, *P* = 0.003). When comparing inter-individual variation (beta diversity), both of the metrics used (Bray–Curtis and weighted UniFrac) clearly reflected the different structure among gilthead seabream from the pre-AMs group and at 24 h post-AMs; while samples from 8 days post-AMs clustered in a different group than the previous ones. In addition, when using Bray–Curtis dissimilarity, which considers the abundance of each species in the community, the gut bacterial communities from gilthead seabream at 8 days post-AMs resembled those of 17 days post-AMs, which were still different to those of fish from the pre-AMs group. Inversely, when also considering the inter-individual phylogenetic relationships among species by the weighted UniFrac distance, the structure of the gut communities from gilthead seabream at 8- and 17-days post-AMs remained different, while the latter was similar to that of the pre-AMs. Thus, the above-mentioned results seemed to indicate that after 17 days the gut microbial diversity, structure and composition was recovered from the AM treatment and were fairly stable. Considering these results, we are confident that by measuring the microbial dynamics during 36 days after the IMT, at the final sampling point we were assessing only the effect of the transplantation in the gut microbiota rather than the long-term effect of the AMs applied to the recipient fish before the transplant.

### Alpha and beta diversity for IMT

The fact that at 36 days post-IMT, gilthead seabream groups fed with the GSB and the salmon diets displayed ACE values that were only similar to those of gilthead seabream from the pre-IMT group and to the Atlantic salmon microbiota, respectively, suggested that the diet was the main driver of the gut bacterial richness. These results are in agreement with previous studies assessing the influence of the diet in humans and fish^55,56^. The impact of the diet was also supported by our beta diversity results. Indeed, at the end of the trial, the gut microbial structure of gilthead seabream was similar to the diet which each group was fed, as shown in weighted UniFrac comparison. On the other hand, Shannon diversity results suggested that the microbial diversity of the fish fed the GSB diet tended to a differentiation from the donor in the medium-term after the transplantation (at 7 days post-IMT)^30^. However, the diversity reached an intermediate state between the donor and recipient diversity in a longer-term, in terms of evenness, already at 16 days post-IMT, and phylogeny, at 36 days post-IMT, as demonstrated by both Shannon and Faith’s phylogenetic diversity indices. In addition, similar to our results, when performing a FMT in yellowtail kingfish (both via gavage or through the water), Legrand et al.^18^ found a modulation at 8–15 days post-FMT towards a reestablishment in the diversity from 0 days post-FMT and from that of their congeners that were not submitted to a FMT. Altogether, our results suggested that the microbiota of gilthead seabream after 36 days from the IMT is the sum of the synergy between the host, the donor, and the administered diet.

### Bacterial community composition of gilthead seabream fed with their homologous (GSB) diet post-IMT

When comparing the bacterial composition of the animals that received the transplanted microbiota and fed with their homologous diet to their initial microbiota from pre-IMT and at 2 days post-IMT, it was notable the emergence of the genera *Lactobacillus* and *Ligilactobacillus*, which showed a very pronounced increase in their relative abundance at the end of the trial with respect to the pre-IMT group. Many species of these lactic acid bacteria (LAB) have been proposed as promising probiotics for humans and in animal production, that can serve as efficient alternatives to antibiotics for improving animal performance and preventing diseases^57,58^. As mentioned above, these genera were not present in the bacterial populations of gilthead seabream from the pre-IMT group nor in the Atlantic salmon microbiota, so it was clear that their origin in the intestine was through their abundant presence in the experimental GSB diet. It is difficult to categorically conclude whether these bacteria colonized the intestinal mucosa of gilthead seabream or were present just in the transient microbiota that circulates through the intestinal tract, or both, since we collected both the autochthonous and allochthonous microbiota in a single analytical sample from each fish. However, their increasing abundance over time seemed to indicate that these probiotic species introduced through the diet were colonizing the gut as an adaptation to a healthy gut condition once the effects of the AM treatment had subsided following the introduction of the IMT. Two other remarkable genera showing significant differences at 2 days post-IMT with respect to gilthead seabream from the pre-IMT group were *Vibrio*, which decreased over time, and *Escherichia-Shigella*, which increased over time. In this case, it was difficult to determine to what extent these differences could be attributed to the influence of the microbial profile from donor Atlantic salmon, to the GSB diet, as it also showed differences for the abundance of these genera with respect to the fish post-IMT at all the times, or to the effect of the AMs, which generated a similar trend in both genera: decrease of *Vibrio* and increase of *Escherichia-Shigella*. These potential pleiotropic differences may also be due to many other factors, such as the thermal impact from the use of Atlantic salmon donors grown at 12 °C rather than the 20 °C at which the seabream were kept, or due to the interaction among gut bacterial communities. As an example of such potential interactions among different bacterial species, LAB and in particular, *Lactobacillus*, are well-known to produce antimicrobial substances that have activity towards many *Vibrio* species^59^, which has been applied to the commercial farming of aquatic species to prevent and control vibriosis^60,61^. Therefore, the reduction of *Vibrio* over time under current conditions, may be partly attributed to the mentioned exponential increase in the abundance of *Lactobacillus* and *Ligilactobacillus*. In the case of *Aliivibrio*, it was clear that its emergence in gilthead seabream (2 days) after the IMT was due to its presence in the donor Atlantic salmon microbiota, as it was not found in the recipient animals before the transplant and in the diet. Some species belonging to this genus are found seasonally in the hindgut of Atlantic salmon^62^. Under current conditions, *Aliivibrio* was not maintained over time, showing relative abundances equal or very close to 0% from 7 days post-IMT onwards, probably due to the water temperature differences between both hosts’ rearing conditions (12 °C in the Atlantic salmon and 20 °C in gilthead seabream). This hypothesis is supported by the fact that many *Aliivibrio* species have an optimal growth rate within the range of 12–18 °C and lose growth capacity at temperatures of approximately 20 °C^63–65^. On the other hand, Legrand et al.^18^ showed a maintenance of various ASVs belonging to the genus *Aliivibrio* when performing a FMT among individuals of yellowtail kingfish reared at 12.7–14 °C at 2 days post-FMT, but these were lost at 8 days post-FMT. This may indicate that this genus, not only to thermal changes, but is also very sensitive to shifts in the environmental conditions. In addition, it is not uncommon to observe that part of the donor bacterial taxa which initially colonized the recipient gut after a FMT are not maintained over time^18,20^. In this sense, a higher efficiency in the persistence of the bacterial composition may be achieved by repeating IMT/FMT over time, as already shown in mice^66^, and considering the higher efficiency of multiple dose-FMTs in humans for therapeutic applications with respect to single dose-FMTs^67–69^. It is also important to note that the current trial was a first conceptual approach in an attempt to understand the microbial dynamics when performing an IMT between two fish spices reared under quite opposite conditions, being in this case the temperature the determining factor, but for greater efficiency in interspecific IMT/FMT, two species cultured at similar conditions must be sought.

At 36 days post-IMT, the results from gilthead seabream fed the GSB diet showed the importance of the homologous diet for defining the fish microbiota in the long-term after an IMT, with 56% of the genera showing a relative abundance similar to the diet. This is consistent with the already demonstrated high proficiency of the diet for determining the bacterial composition of the fish gut during non-starvation periods^56,70^. The microbiota from the Atlantic salmon and baseline gilthead seabream from the pre-IMT group also played an important, but slighter, role in shaping the gut bacterial communities (31% of the genera had a relative abundance similar to gilthead seabream from the pre-IMT group and Atlantic salmon microbiota). These results were partly in agreement with the previous studies of microbial transplants that have been performed in fish. In particular, while Smith et al.^15^ and Zhang et al.^17^ showed successful results for African turquoise killifish and large yellow croaker respectively, their microbial composition in terms of phyla and genera and their abundances varied with respect to both the donor and recipient animals. On the other hand, in the work of Legrand et al.^18^ in yellowtail kingfish microbiota, the FMT only worked in some individuals, and in any case, the transplanted animals displayed a composition profile much more similar to the recipient fish than to the donors. Regarding microbial transplants among different species, when Rawls et al.^21^ transplanted the gut microbiota of mouse into germ-free zebrafish, the vast majority of phyla from the donor mice were maintained, adapting their abundances to the recipient animals. However, few genera from the donors were maintained after the transference, similar to what was observed in the study of Valenzuela et al.^20^ after performing a FMT from human to zebrafish. In line with this, Toh et al.^19^ performed an inoculation of bacteria isolated from cultures derived from the human gastrointestinal tract into zebrafish, but only two out of 22 species were established in the zebrafish gut microbiota. Nonetheless, our study is the first attempt to perform an IMT between two different fish species and none of the above-mentioned studies considered to evaluate the impact of the diet on the microbiota of the transplanted fish. In this sense, in the present work, at the final sampling point the effect of the bacterial composition from the diet was clear for the relative abundances of *Peptostreptococcus* and *Limosilactobacillus*, as these genera found in the GSB diet were not present in the Atlantic salmon microbiota nor in the gilthead seabream from the pre-IMT group. Another genus which was probably influenced by the diet over time was *Photobacterium*, since the presence of *Lactobacillus,* either present in the diet as a probiotic or in the fish intestine, has been suggested to negatively regulate the abundance of *Photobacterium* in shrimp^71^ and fish species such as gilthead seabream^72^, in the same manner as described above for *Vibrio*. Related to this, some species belonging to the Vibrionaceae family (*Photobacterium* and *Vibrio* genera) may act as potential pathogens^54^, so it may be hypothesized that the microbial transplant executed within the present assay, and the homologous experimental diet provided to the recipient fish thereafter, may have a beneficial effect on the host’s health by the exclusion of potential pathogenic bacteria. However, the genera *Photobacterium* and *Vibrio* also contain many species that are typical symbionts in fish intestines that can produce hydrolytic enzymes that help with digestion^39,54^, so for demonstrating their effect on the host intestinal health further studies focused on characterizing these species and their metabolic potentials would be needed.

Apart from the impact of the diet during the trial, these results showed that the initial gilthead seabream microbiota (pre-AMs and pre-IMT) also had an important influence in the short-term establishment of the microbiota after the IMT. This was reflected in the presence of *Catenococcus* and *Brevinema* up to 16 days post-IMT, as both genera were found only in gilthead seabream from the pre-IMT group, but not in the Atlantic salmon microbiota, nor in the GSB diet. Therefore, their presence after the IMT may be associated to either environmental factors or to factors intrinsic to the fish species such as their physiology, or genetic background, as both bacterial genera have been previously reported as part of the autochthonous fish microbiota^72–74^. Neither of these genera were maintained in the long-term (36 days post-IMT), though it may be due to the higher impact of the GSB diet, as the abundance of *Brevinema* in the fish gut mucosa has been shown to be easily modulated by the ingredient composition of the diet^75,76^, or due to competition with other bacteria after the IMT. Indeed, the competition among bacterial populations and their subsequent decrease in abundance of some may free up gut niches and resources needed for the emergence and growth of novel bacterial species, such as those belonging to the genera *Acinetobacter* and *Corynebacterium*, which were not present in the gut microbiota of Atlantic salmon or gilthead seabream from the pre-IMT group, nor in the GSB diet. Curiously, a previous assay in Atlantic salmon has shown a correlation between the increase of rearing water temperatures up to 21 °C and the disappearance of *Acinetobacter* and LAB, and increase of *Vibrio*^77^. Thus, under current experimental conditions the above-mentioned differences in the abundance of these genera before and after the IMT may also be related to microbial communities coming from fish normally restricted to lower water temperatures (12 °C) being introduced into an environment with higher temperatures (20 °C). However, interestingly, in the present study, the observed dynamics were opposite to those that would be expected based on the work of the former authors, which indicated that some bacteria have the capacity to adapt to new conditions, which supports the relevance and viability of performing interspecific IMTs^21,78^. In this sense, it is well-known that the fish microbiota is a constantly changing element, which has the capacity to coevolve with the host and create very unique adaptations that are not found in other vertebrate groups^79,80^. There are many ways the holobiont may be influenced, and the microbial communities within can adapt, thereby altering the holobiont. Such complexity of the holobiont is clearly reflected in the difficulty to elucidate what are the primary drivers altering the composition of the microbiota, whether by the composition of the donors, recipients, or by the diet, as exemplified in the current study. In addition, there are few assays performing inter-species microbiota transfers in fish^19–21^ and none of them had considered the diet as a key factor, until now. More studies of this type will be needed to gain better knowledge about the modulation of the fish gut microbiota through host-, donor-, and diet-microbiota interactions within the holobiont.

### Bacterial community composition of gilthead seabream fed with their heterologous (salmon) diet post-IMT

Similar to the gilthead seabream subjected to the IMT and then fed with their homologous diet, at 2 days post-IMT in the fish fed the heterologous diet (for salmon) the abundances of all represented phyla were similar to those observed in the Atlantic salmon microbiota. On the other hand, although most phyla also presented a relative abundance like that of gilthead seabream from the pre-IMT group, Bacteroidota increased significantly after the IMT, possibly due to the effect of AMs that boosted their abundance, or due to their significant abundance in the salmon diet, as compared to the lower value in the GSB diet. At the genus level, at 2 days post-IMT the decreased abundance in *Vibrio* and increase in *Escherichia-Shigella*, with respect to gilthead seabream from the pre-IMT group, evinced that the effects of the AMs were mainly responsible for such changes in abundance in the above mentioned genera, as these same dynamics were also observed in the other two groups (gilthead seabream fed with the GSB diet and for those that were fasted) shortly after the AM treatment. These results were partly expected since some *Vibrio* strains have been shown to be sensitive to both ampicillin and neomycin (both included in the AM cocktail)^81–83^. In addition, *Escherichia-Shigella* also showed an increase in the gut of mice treated with an antibiotic cocktail containing the same antibiotics that we used^41^. Furthermore, the decrease in *Vibrio* and increase in *Escherichia-Shigella* were maintained in all sampling times post-IMT, with the latter showing an increasing colonization of gilthead seabream gut over time. *Escherichia-Shigella* is habitually found in the intestine of farmed gilthead seabream^75,84^, and have been reported as part of the core gut microbiota in some fish species^85–87^; thus, its constant increasing abundance may be attributed to its survival and persistent growth over time as part of the gilthead seabream core microbiota. The persistency of the core microbiota may be consistent with the high abundance of the ASVs which were shared among gilthead seabream fed the GSB and the salmon diets and the group not submitted to the IMT (post-AMs) at both 7 and 16 days post-IMT, as shown in the Venn diagram (Fig. 9E,F). In addition, the fact that there were no significant differences in beta diversity among fasted gilthead seabream at 17 days post-AMs and the group fed the GSB diet at 16 days post-IMT suggested a progression towards a common status in both groups once the microbial communities are recovered from the AM treatment and adapted to the changes caused by the transplant. However, this was not observed in the group fed the salmon diet, which showed significant differences with respect to fasted gilthead seabream at 17 days post-AMs, underlining again the essential role of the diet in the definition of the microbiota, as discussed above.

On the other hand, the up-regulation of *Photobacterium* at 2 days after the IMT seemed to be mainly a direct consequence of the abundant presence of this genus in the gut microbiota of donor fish, which may be also increased by its presence in the gut of the recipient gilthead seabream. At this point, the effect of the salmon diet in the modulation of the abundance of *Photobacterium* was probably irrelevant as only 2 days had passed since the IMT and its presence in the diet was very low. However, over a longer-term scope, the initial high density of this genus in donor and recipient fishes apparently moved to the background, and it might be plausible that other factors such as the diet took priority. In this sense, as happened with fish fed the GSB diet, the decrease of *Vibrio* and *Photobacterium* over time might have been a response to the presence of LAB in the feed, albeit at a lower percentage compared to the GSB diet. However, for fish fed the salmon diet, the LAB did not ultimately colonize the intestinal mucosa (absence of both genera at 36 days post), but these bacteria were included in the diet being fed, and bacterial colonization is well-known to not be necessary to induce host microbiota modification^88–90^. On the other hand, the increased abundance of *Aliivibrio* at 2 days after the IMT was clearly a consequence of its abundance in the Atlantic salmon microbiota, as it was present neither in the recipient fish microbiota nor in the salmon diet, and again its disappearance over time may be related to the change of environmental temperatures. A remarkable appearance of *Mycoplasma* at 7 days post-IMT was noted, which was only present in the Atlantic salmon microbiota and is commonly found in the intestinal mucosa of salmonids^91^, thus indicating that the *Mycoplasma* likely originated from the donor microbiota. Interestingly, when the relative abundance of *Mycoplasma* increased at 7 days post-IMT, that of *Aliivibrio* decreased to 0%, in accordance with the model proposed by Scheuring et al.^92^, which suggested resource competition between these two genera and toxicity of some *Aliivibrio* strains towards *Mycoplasma*. However, *Aliivibrio* did not remain over time, as its abundance was very low and probably there were not optimal growing conditions for species of this genus, or the competition with other bacteria prevented their growth.

The influence of the three tested factors (feed, donor microbiota and recipient microbiota) on the establishment of the gut bacterial communities after an IMT was much higher in the case of fish fed the salmon diet than in their congeners fed the GSB diet. In particular, 77% of the genera were similar to gilthead seabream from the pre-IMT group and Atlantic salmon microbiota, while 65% of genera presented a relative abundance similar to the microbial profile of the salmon diet. Apart from *Photobacterium*, *Vibrio* and *Escherichia-Shigella*, the only genera that showed significant differences at the final sampling point, with respect to gilthead seabream from the pre-IMT group, were: *Acinetobacter*, *Catenococcus* and *Vibrionimonas*. Regarding *Acinetobacter*, probably as happened with *Pseudomonas*, there was a colonization of the gut mucosa by these genera from dietary origin. Both genera from the order Pseudomonadales tend to inhabit the intestinal mucosa of fish^93^ and have been reported to possess enzymatic activities that favour a higher nutrient digestion^94^. The Pseudomonadales are also noted for their ability to metabolize a wide variety of substrates, including various antibiotics^45^, which may explain their higher plasticity to grow out in fish gut after the AM treatment. This metabolic activity in the current context, would give those bacterial species an added benefit towards survival during the initial stages of this trial, as seen in the fasted gilthead seabream with the increased relative abundance of *Acinetobacter* and *Pseudomonas* at 24 h post-AMs. In gilthead seabream fed the salmon diet after the IMT, some genera which were present at lower abundances in the salmon diet (≤ 0.5%), and were not present in the donor’s microbiota, nor in gilthead seabream from the pre-IMT group showed a similar trend to that shown for the Pseudomonodales. However, such genera showed greater abundances at the final sampling time: *Vibrionimonas*, *Corynebacterium*, *Sphingomonas*, and *Cutibacterium*. As *Acinetobacter* and *Corynebacterium* also appeared at 36 days post-IMT (though at lower %) in fish fed the GSB diet (which were not part of the core microbiota of the recipient fish), the abundances of the mentioned genera in the fish gut may not only be attributed to their dietary origin. However, other hypotheses may not be excluded such as the emergence of some strains, possibly induced by specific compounds provided in the diet or by other factors, like the competition among bacteria for resources after the IMT. Finally, unlike fish fed the GSB diet, the feeding of the salmon diet led to the incorporation of *Catenococcus* through the diet and this genus was maintained until 36 days post-IMT, reaffirming that the microbial composition of the diet is a fundamental element in defining the microbiota after a dysbiosis caused by such an invasive strategy as both anal and oral antibiotic gavage followed by an IMT.

## Conclusions

The methodological approach for performing an intestinal microbiota transplant between fish as outlined in the current study sheds light on the mechanisms underlying the modulation of the host microbiota after a gut microbial transplant between different fish species, with the added value that it not only considers the microbiota and the different environmental conditions of the donor and the recipient fishes, but also the bacteria present in the diets of donor and recipient. Among our findings, the effect of the diet type was notable for defining the gut estimated richness (ACE index) in the long-term and the bacterial diversity (Shannon) and phylogenetic diversity (Faith index) in the short-term, that reached an equilibrium inherent to the host environment or to the animal over the longer term. The diet displayed a high level of influence in defining the gut microbial structure, as reflected by the weighted UniFrac values. Regarding composition, it was difficult to assess to what extent the similarities or differences in abundances found over time after the IMT were due to the donor and recipient fish microbiota, to the bacteria present in the feed, or to the summation of all of them, since there are many factors which indirectly may affect bacterial abundance, such as the change of environmental conditions (i.e., inhibition of growth at certain environmental conditions for *Aliivibrio*), competition among bacteria (i.e., inhibition of *Photobacterium* and *Vibrio* by LAB, or competition between *Aliivibrio* and *Mycoplasma*), release of metabolites to the gut environment (i.e., toxins by *Aliivibrio*), etc. What can undoubtedly be drawn from this study is that: (i) the “core” microbiota of the animal is capable of being reestablished over time in the absence of outside influence such as diet, despite subjecting the host to different dysbiosis causing strategies such as a treatment of AM or an IMT; (ii) the diet plays a very important role in defining the gut microbiota of the host animal, as seen in those gilthead seabream fed the GSB diet, where the bacterial composition more closely resembled that of the feed; and (iii) in fish fed the salmon diet, while there was a large part of the bacterial taxa composition from the Atlantic salmon gut microbiota that was also maintained or reestablished by the end of the assay, the overall microbiota was more unique arising from a combination of extrinsic and intrinsic drivers. From a future perspective, there exists some potential for application of IMT for improvement of digestive capabilities and enhancement of fish health that is worthy of future exploration. In this sense, microbial transplants have been shown to be a good strategy to improve growth and feed performance, not only in ruminants^13,95^, but also in swine^96^ and, slightly, in poultry^97^. Overall, the application of IMT in fish seems to be a promising strategy with multitude of potential applications to be developed, but for that, in parallel with this, it is important to continue evaluating the impact of the different factors affecting the gut microbial composition and functionality when applying this strategy. However, it is also important to consider that a limiting factor of IMTs in fish is the number of individuals that are managed in aquaculture, which makes their massive application on an industrial scale difficult.

### Supplementary Information

Supplementary Figure 1.

Supplementary Figure 2.

Supplementary Figure 3.

Supplementary Figure 4.

Supplementary Figure 5.

Supplementary Table 1.

Supplementary Table 2.

Supplementary Table 3.

Supplementary Table 4.

Supplementary Table 5.

Supplementary Table 6.

## Data Availability

Raw sequencing data and metadata for all samples included in this study have been uploaded to the Sequence Read Archive (SRA) database of NCBI (https://www.ncbi.nlm.nih.gov/sra ) and can be accessed with the Bioproject accession number PRJNA1029014, while the processed data outcomes have been included within the manuscript or additional files.
