# Supplementary material for: Impact of the diet in the gut microbiota after an inter-species microbial transplantation in fish
Source: Sci Rep. 2024 Feb 18;14:4007. doi: 10.1038/s41598-024-54519-6 (PMC10874947; doi:10.1038/s41598-024-54519-6)

**Fig S1.** Rarefaction curves represented in function of observed ASVs with respect to the number of reads for the samples used to estimate alpha diversity.


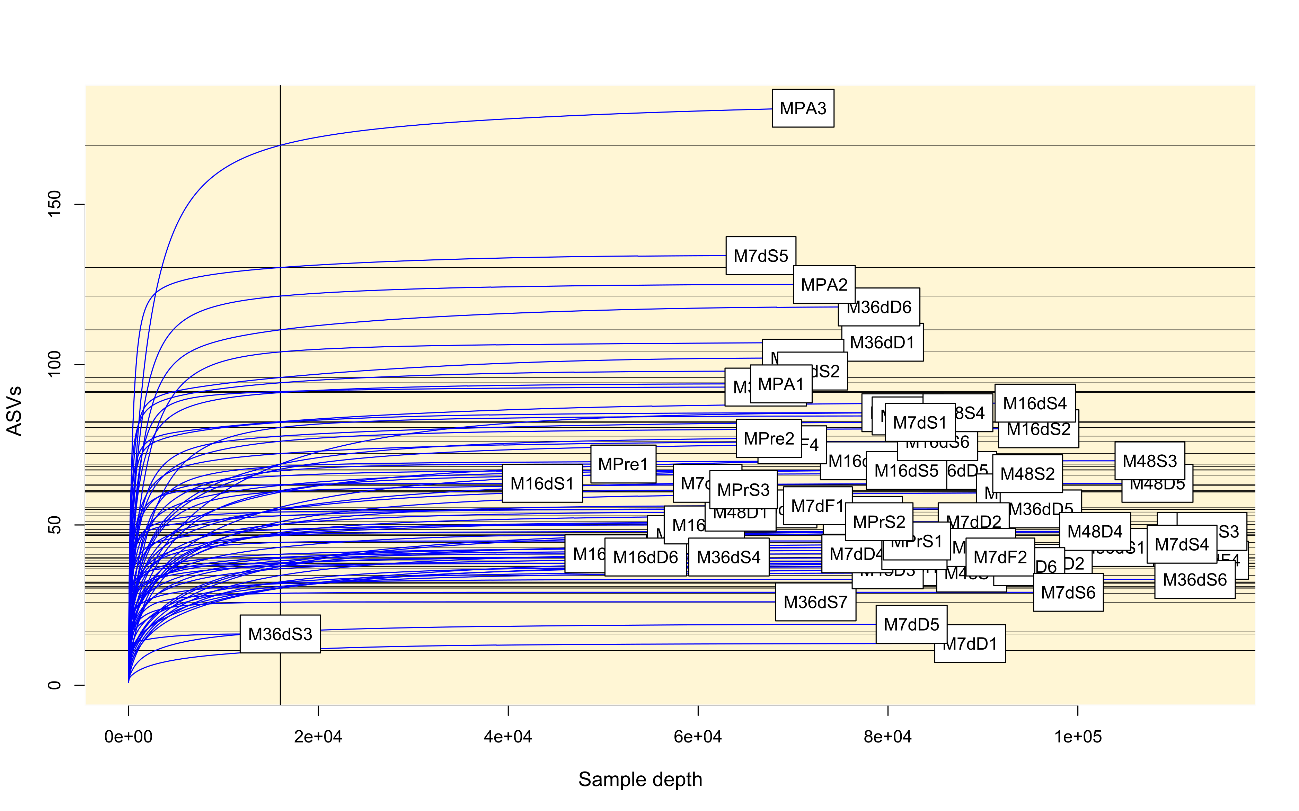

Supplement: Supplementary file 1 — Supplementary Figure 1. [file 41598_2024_54519_MOESM1_ESM.docx]
