# Supplementary material for: Impact of the diet in the gut microbiota after an inter-species microbial transplantation in fish
Source: Sci Rep. 2024 Feb 18;14:4007. doi: 10.1038/s41598-024-54519-6 (PMC10874947; doi:10.1038/s41598-024-54519-6)
Supplement: Supplementary file 3 — Supplementary Figure 3. [file 41598_2024_54519_MOESM3_ESM.docx]

**Fig. S3.** Significant differences on the relative abundances of genera from the gut bacterial communities ≥ 0.5% among gilthead seabream fed their typical GSB diet at 2, 7, 16 and 36 days post-IMT with respect to gilthead seabream diet (GSB diet), Atlantic salmon (microbiota donor), and gilthead seabream previous to the intestinal microbiota transplant (GSB pre-IMT). *P*-values from Kruskal-Wallis test are indicated in bold, while significant differences in Wilcoxon *post-hoc* test are indicated in red (*P* ≤ 0.1) and absence of differences in green (*P* > 0.1).
