# Supplementary material for: Impact of the diet in the gut microbiota after an inter-species microbial transplantation in fish
Source: Sci Rep. 2024 Feb 18;14:4007. doi: 10.1038/s41598-024-54519-6 (PMC10874947; doi:10.1038/s41598-024-54519-6)
Supplement: Supplementary file 6 — Supplementary Table 1. [file 41598_2024_54519_MOESM6_ESM.docx]

**Table S1.** Relative abundances of phyla from the gut bacterial communities ≥ 0.5% in gilthead seabream pre-antimicrobials (GSB pre-AMs) and at 24 h, 8 days and 17 days post-antimicrobials (post-AMs).

|  | ***P*-value** | **GSB pre-AMs** | **GSB 24 h post-AMs** | **GSB 8 days post-AMs** | **GSB 17 days post-AMs** |
| --- | --- | --- | --- | --- | --- |
| **Proteobacteria** | 0.057 | 89.17 ± 3.97^b^ | 38.68 ± 0.59^a^ | 88.76 ± 6.49^b^ | 84.24 ± 5.39^b^ |
| **Firmicutes** | 0.017 | 5.13 ± 1.06^b^ | 12.34 ± 3.06^c^ | 1.48 ± 1.47^a^ | 3.77 ± 3.15^ab^ |
| **Cyanobacteria** | 0.01 | 0.00 ± 0.00^a^ | 12.51 ± 2.15^c^ | 3.91 ± 2.37^b^ | 2.21 ± 0.99^b^ |
| **Actinobacteriota** | 0.027 | 0.00 ± 0.00^a^ | 13.76 ± 1.47^c^ | 2.26 ± 2.63^b^ | 1.12 ± 1.35^ab^ |
| **Unassigned** | 0.115 | 5.25 ± 4.60 | 3.21 ± 2.30 | 0.33 ± 0.45 | 4.41 ± 4.16 |
| **Planctomycetota** | 0.029 | 0.00 ± 0.00^a^ | 7.09 ± 0.54^b^ | 1.27 ± 1.26^a^ | 0.95 ± 1.23^a^ |
| **Spirochaetota** | 0.535 | 0.45 ± 0.40 | 0.99 ± 0.16 | 1.09 ± 1.53 | 1.66 ± 1.15 |
| **Verrucomicrobiota** | 0.027 | 0.00 ± 0.00^a^ | 3.83 ± 1.29^b^ | 0.29 ± 0.40^a^ | 0.62 ± 0.73^a^ |
| **Bacteroidota** | 0.01 | 0.00 ± 0.00^a^ | 2.74 ± 0.50^b^ | 0.00 ± 0.00^a^ | 0.65 ± 0.81^a^ |
| **Dependentiae** | 0.003 | 0.00 ± 0.00^a^ | 2.62 ± 0.45^b^ | 0.00 ± 0.00^a^ | 0.00 ± 0.00^a^ |

Values are represented as mean ± SD. Significant differences among experimental groups are indicated by the different superscript letters (Kruskal-Wallis with Wilcoxon *post-hoc* test; *P* ≤ 0.1).
