# Supplementary material for: Impact of the diet in the gut microbiota after an inter-species microbial transplantation in fish
Source: Sci Rep. 2024 Feb 18;14:4007. doi: 10.1038/s41598-024-54519-6 (PMC10874947; doi:10.1038/s41598-024-54519-6)
Supplement: Supplementary file 7 — Supplementary Table 2. [file 41598_2024_54519_MOESM7_ESM.docx]

**Table S2.** Relative abundances of genera from the gut bacterial communities ≥ 0.5% in gilthead seabream pre-antimicrobials (GSB pre-AMs) and at 24 h, 8 days and 17 days post-antimicrobials (post-AMs).

|  | ***P*-value** | **GSB pre-AMs** | **GSB 24 h**  **post-AMs** | **GSB 8 days**  **post-AMs** | **GSB 17 days**  **post-AMs** |
| --- | --- | --- | --- | --- | --- |
| **Proteobacteria\|Gammaproteobacteria\|Enterobacterales\|Vibrionaceae\|*Vibrio*** | 0.012 | 56.21 ± 7.75^c^ | 2.92 ± 0.26^a^ | 20.33 ± 11.77^b^ | 19.16 ± 15.59^b^ |
| **Proteobacteria\|Gammaproteobacteria\|Enterobacterales\|Vibrionaceae\|*Photobacterium*** | 0.084 | 12.66 ± 3.08^ab^ | 9.38 ± 3.47^a^ | 19.52 ± 8.31^b^ | 16.09 ± 3.35^ab^ |
| **Proteobacteria\|Gammaproteobacteria\|Enterobacterales\|Vibrionaceae\|Unassigned** | 0.019 | 10.10 ± 1.53^b^ | 1.59 ± 0.72^a^ | 12.90 ± 9.06^bc^ | 19.65 ± 9.82^c^ |
| **Proteobacteria\|Gammaproteobacteria\|Enterobacterales\|Vibrionaceae\|*****Aliivibrio*** | 0.035 | 0.00 ± 0.00^a^ | 0.00 ± 0.00^a^ | 17.63 ± 20.51^b^ | 7.08 ± 9.62^ab^ |
| **Proteobacteria\|Gammaproteobacteria\|Enterobacterales\|Vibrionaceae\|*Catenococcus*** | 0.177 | 6.73 ± 1.08 | 4.38 ± 0.83 | 7.29 ± 3.62 | 8.85 ± 7.13 |
| **Unassigned\|Unassigned\|Unassigned\|Unassigned\|Unassigned** | 0.115 | 5.25 ± 4.60 | 3.21 ± 2.30 | 0.33 ± 0.45 | 4.41 ± 4.16 |
| **Cyanobacteria\|Cyanobacteriia\|Synechococcales\|Cyanobiaceae\|*****Synechococcus* CC9902** | 0.011 | 0.00 ± 0.00^a^ | 8.71 ± 2.43^c^ | 2.68 ± 1.37^b^ | 1.24 ± 0.87^ab^ |
| **Proteobacteria\|Gammaproteobacteria\|Enterobacterales\|Enterobacteriaceae\|*****Escherichia-Shigella*** | 0.045 | 0.51 ± 0.45^a^ | 2.30 ± 0.46^b^ | 1.86 ± 0.43^b^ | 2.15 ± 0.61^b^ |
| **Cyanobacteria\|Cyanobacteriia\|Synechococcales\|Cyanobiaceae\|*****Cyanobium* PCC-6307** | 0.027 | 0.00 ± 0.00^a^ | 3.31 ± 0.47^b^ | 0.81 ± 0.74^a^ | 0.97 ± 0.68^a^ |
| **Firmicutes\|Bacilli\|Staphylococcales\|Staphylococcaceae\|*****Staphylococcus*** | 0.027 | 0.00 ± 0.00^a^ | 3.94 ± 0.28^b^ | 0.58 ± 0.82^a^ | 0.75 ± 1.51^a^ |
| **Planctomycetota\|Planctomycetes\|Pirellulales\|****Pirellulaceae\|Unassigned** | 0.027 | 0.00 ± 0.00^a^ | 3.89 ± 0.43^b^ | 0.81 ± 0.83^a^ | 0.48 ± 0.59^a^ |
| **Spirochaetota\|Brevinematia\|Brevinematales\|Brevinemataceae\|*Brevinema*** | 0.535 | 0.45 ± 0.40 | 0.99 ± 0.16 | 1.09 ± 1.53 | 1.66 ± 1.15 |
| **Firmicutes\|Clostridia\|Clostridiales\|Clostridiaceae\|Unassigned** | 0.009 | 4.78 ± 0.83^b^ | 0.00 ± 0.00^a^ | 0.00 ± 0.00^a^ | 0.34 ± 0.67^a^ |
| **Proteobacteria\|Gammaproteobacteria\|Unassigned\|Unassigned\|Unassigned** | 0.149 | 0.00 ± 0.00 | 1.68 ± 0.57 | 0.64 ± 1.08 | 1.30 ± 1.00 |
| **Proteobacteria\|Gammaproteobacteria\|Pseudomonadales\|Moraxellaceae\|*****Acinetobacter*** | 0.099 | 0.00 ± 0.00^a^ | 0.64 ± 0.13^b^ | 0.90 ± 0.69^b^ | 1.71 ± 1.32^ab^ |
| **Firmicutes\|Bacilli\|Bacillales\|Unassigned\|Unassigned** | 0.046 | 0.00 ± 0.00^a^ | 2.36 ± 1.21^b^ | 0.23 ± 0.51^a^ | 0.49 ± 0.58^ab^ |
| **Actinobacteriota\|Actinobacteria\|PeM15\|Unassigned\|Unassigned** | 0.024 | 0.00 ± 0.00^a^ | 2.31 ± 0.12^b^ | 0.46 ± 0.64^a^ | 0.23 ± 0.46^a^ |
| **Proteobacteria\|Gammaproteobacteria\|Coxiellales\|Coxiellaceae\|*****Coxiella*** | 0.03 | 0.00 ± 0.00^a^ | 2.30 ± 1.22^b^ | 0.14 ± 0.31^a^ | 0.36 ± 0.73^a^ |
| **Actinobacteriota\|Actinobacteria\|Micrococcales\|Microbacteriaceae\|DS001** | 0.048 | 0.00 ± 0.00^a^ | 1.80 ± 0.65^b^ | 0.70 ± 0.96^ab^ | 0.00 ± 0.00^a^ |
| **Firmicutes\|Bacilli\|Lactobacillales\|Streptococcaceae\|*****Streptococcus*** | 0.062 | 0.00 ± 0.00^a^ | 1.64 ± 0.40^b^ | 0.19 ± 0.42^a^ | 0.57 ± 1.13^ab^ |

Values are represented as mean ± SD. Significant differences among experimental groups are indicated by the different superscript letters (Kruskal-Wallis with Wilcoxon *post-hoc* test; *P* ≤ 0.1).
