# Supplementary material for: Impact of the diet in the gut microbiota after an inter-species microbial transplantation in fish
Source: Sci Rep. 2024 Feb 18;14:4007. doi: 10.1038/s41598-024-54519-6 (PMC10874947; doi:10.1038/s41598-024-54519-6)
Supplement: Supplementary file 8 — Supplementary Table 3. [file 41598_2024_54519_MOESM8_ESM.docx]

**Table S3.** Relative abundances of phyla from the gut bacterial communities ≥ 0.5% in gilthead seabream diet (GSB diet), Atlantic salmon (microbiota donor), gilthead seabream previous to the intestinal microbiota transplant (GSB pre-IMT) and in gilthead seabream fed their typical GSB diet at 2, 7, 16 and 36 days post-IMT.

|  | **GSB diet** | **Salmon** | **GSB pre-IMT** | **GSB 2 days**  **post-IMT** | **GSB 7 days**  **post-IMT** | **GSB 16 days**  **post-IMT** | **GSB 36 days**  **post-IMT** |
| --- | --- | --- | --- | --- | --- | --- | --- |
| **Proteobacteria** | 15.52 ± 0.94 | 93.37 ± 1.70 | 89.17 ± 3.97 | 87.73 ± 11.90 | 48.99 ± 7.41 | 77.86 ± 14.37 | 13.02 ± 4.96 |
| **Firmicutes** | 68.32 ± 6.90 | 5.87 ± 2.24 | 5.13 ± 1.06 | 4.41 ± 3.50 | 5.75 ± 4.94 | 10.11 ± 7.25 | 77.17 ± 4.56 |
| **Spirochaetota** | 0.00 ± 0.00 | 0.00 ± 0.00 | 0.45 ± 0.40 | 0.71 ± 0.97 | 27.44 ± 13.62 | 3.63 ± 3.15 | 0.00 ± 0.00 |
| **Actinobacteriota** | 0.00 ± 0.00 | 0.18 ± 0.32 | 0.00 ± 0.00 | 1.35 ± 1.96 | 3.76 ± 4.26 | 1.77 ± 1.69 | 6.48 ± 1.81 |
| **Bacteroidota** | 3.35 ± 4.08 | 0.18 ± 0.30 | 0.00 ± 0.00 | 1.14 ± 1.81 | 7.24 ± 5.99 | 1.07 ± 1.57 | 0.62 ± 0.95 |
| **Cyanobacteria** | 0.00 ± 0.00 | 0.00 ± 0.00 | 0.00 ± 0.00 | 1.97 ± 2.86 | 2.17 ± 2.10 | 2.70 ± 2.86 | 1.56 ± 1.20 |
| **Unassigned** | 0.87 ± 1.51 | 0.00 ± 0.00 | 5.25 ± 4.60 | 1.44 ± 3.22 | 1.47 ± 0.90 | 1.94 ± 3.58 | 0.19 ± 0.20 |
| **Fusobacteriota** | 11.93 ± 3.91 | 0.00 ± 0.00 | 0.00 ± 0.00 | 0.00 ± 0.00 | 0.00 ± 0.00 | 0.00 ± 0.00 | 0.16 ± 0.43 |

Values are represented as mean ± SD.
