# Supplementary material for: Impact of the diet in the gut microbiota after an inter-species microbial transplantation in fish
Source: Sci Rep. 2024 Feb 18;14:4007. doi: 10.1038/s41598-024-54519-6 (PMC10874947; doi:10.1038/s41598-024-54519-6)
Supplement: Supplementary file 9 — Supplementary Table 4. [file 41598_2024_54519_MOESM9_ESM.docx]

**Table S4.** Relative abundances of genera from the gut bacterial communities ≥ 0.5% in gilthead seabream diet (GSB diet), Atlantic salmon (microbiota donor), gilthead seabream previous to the intestinal microbiota transplant (GSB pre-IMT) and in gilthead seabream fed their typical GSB diet at 2, 7, 16 and 36 days post-IMT.

|  | **GSB diet** | **Salmon** | **GSB pre-IMT** | **GSB 2 days post-IMT** | **GSB 7 days post-IMT** | **GSB 16 days post-IMT** | **GSB 36 days post-IMT** |
| --- | --- | --- | --- | --- | --- | --- | --- |
| **Proteobacteria\|Gammaproteobacteria\|Enterobacterales\|Vibrionaceae\|*Vibrio*** | 1.98 ± 3.43 | 10.26 ± 0.38 | 56.21 ± 7.75 | 19.08 ± 7.27 | 4.31 ± 4.61 | 31.23 ± 14.64 | 0.00 ± 0.00 |
| **Proteobacteria\|Gammaproteobacteria\|Enterobacterales\|Vibrionaceae\|*Photobacterium*** | 7.85 ± 2.82 | 7.18 ± 0.68 | 12.66 ± 3.08 | 29.23 ± 10.08 | 4.31 ± 6.99 | 15.14 ± 5.33 | 0.43 ± 0.35 |
| **Firmicutes\|Bacilli\|Lactobacillales\|Lactobacillaceae\|*Lactobacillus*** | 30.45 ± 5.06 | 0.00 ± 0.00 | 0.00 ± 0.00 | 1.50 ± 0.92 | 2.11 ± 4.19 | 3.69 ± 2.97 | 22.45 ± 6.90 |
| **Firmicutes\|Bacilli\|Lactobacillales\|Lactobacillaceae\|*Ligilactobacillus*** | 14.84 ± 2.64 | 0.00 ± 0.00 | 0.00 ± 0.00 | 1.41 ± 0.87 | 1.16 ± 1.82 | 2.32 ± 1.37 | 27.63 ± 6.57 |
| **Proteobacteria\|Gammaproteobacteria\|Enterobacterales\|Vibrionaceae\|Unassigned** | 0.00 ± 0.00 | 13.79 ± 0.23 | 10.10 ± 1.53 | 13.45 ± 2.40 | 0.53 ± 1.30 | 13.03 ± 6.66 | 0.00 ± 0.00 |
| **Proteobacteria\|Gammaproteobacteria\|Enterobacterales\|Vibrionaceae\|*Aliivibrio*** | 0.00 ± 0.00 | 57.59 ± 5.09 | 0.00 ± 0.00 | 5.53 ± 4.14 | 0.00 ± 0.00 | 0.28 ± 0.69 | 0.11 ± 0.21 |
| **Spirochaetota\|Brevinematia\|Brevinematales\|Brevinemataceae\|*Brevinema*** | 0.00 ± 0.00 | 0.00 ± 0.00 | 0.45 ± 0.40 | 0.71 ± 0.97 | 27.44 ± 13.62 | 3.63 ± 3.15 | 0.00 ± 0.00 |
| **Proteobacteria\|Gammaproteobacteria\|Enterobacterales\|Vibrionaceae\|*Catenococcus*** | 0.00 ± 0.00 | 0.00 ± 0.00 | 6.73 ± 1.08 | 9.58 ± 2.68 | 3.88 ± 3.25 | 7.91 ± 2.40 | 0.00 ± 0.00 |
| **Proteobacteria\|Gammaproteobacteria\|Enterobacterales\|Enterobacteriaceae\|*Escherichia-Shigella*** | 0.00 ± 0.00 | 0.68 ± 0.62 | 0.51 ± 0.45 | 2.16 ± 0.62 | 7.21 ± 6.42 | 2.17 ± 0.61 | 4.03 ± 4.16 |
| **Firmicutes\|Bacilli\|Lactobacillales\|Lactobacillaceae\|Unassigned** | 5.92 ± 1.17 | 0.00 ± 0.00 | 0.00 ± 0.00 | 0.00 ± 0.00 | 0.00 ± 0.00 | 0.68 ± 0.74 | 5.24 ± 2.25 |
| **Unassigned\|Unassigned\|Unassigned\|Unassigned\|Unassigned** | 0.87 ± 1.51 | 0.00 ± 0.00 | 5.25 ± 4.60 | 1.44 ± 3.22 | 1.47 ± 0.90 | 1.94 ± 3.58 | 0.19 ± 0.20 |
| **Proteobacteria\|Gammaproteobacteria\|Burkholderiales\|Comamonadaceae\|Unassigned** | 0.00 ± 0.00 | 0.00 ± 0.00 | 0.00 ± 0.00 | 1.48 ± 1.41 | 5.80 ± 5.45 | 0.54 ± 1.32 | 0.21 ± 0.57 |
| **Proteobacteria\|Gammaproteobacteria\|Pseudomonadales\|Moraxellaceae\|*Acinetobacter*** | 0.00 ± 0.00 | 0.00 ± 0.00 | 0.00 ± 0.00 | 0.52 ± 1.16 | 3.21 ± 3.14 | 1.28 ± 1.68 | 2.29 ± 0.59 |
| **Cyanobacteria\|Cyanobacteriia\|Synechococcales\|Cyanobiaceae\|*Synechococcus* CC9902** | 0.00 ± 0.00 | 0.00 ± 0.00 | 0.00 ± 0.00 | 1.39 ± 2.16 | 1.15 ± 2.05 | 1.75 ± 1.78 | 1.31 ± 1.30 |
| **Actinobacteriota\|Actinobacteria\|Corynebacteriales\|Corynebacteriaceae\|*Corynebacterium*** | 0.00 ± 0.00 | 0.00 ± 0.00 | 0.00 ± 0.00 | 0.00 ± 0.00 | 1.18 ± 1.37 | 0.19 ± 0.47 | 2.45 ± 1.32 |
| **Proteobacteria\|Alphaproteobacteria\|Rhizobiales\|Xanthobacteraceae\|Unassigned** | 0.00 ± 0.00 | 0.00 ± 0.00 | 0.00 ± 0.00 | 0.86 ± 0.79 | 2.35 ± 2.74 | 0.42 ± 1.02 | 0.59 ± 1.13 |
| **Firmicutes\|Bacilli\|Bacillales\|Bacillaceae\|Unassigned** | 0.96 ± 1.66 | 0.00 ± 0.00 | 0.00 ± 0.00 | 0.00 ± 0.00 | 0.30 ± 0.73 | 0.73 ± 0.87 | 2.22 ± 1.67 |
| **Firmicutes\|Clostridia\|Peptostreptococcales-Tissierellales\|Peptostreptococcaceae\|*Peptostreptococcus*** | 1.16 ± 2.02 | 0.00 ± 0.00 | 0.00 ± 0.00 | 0.00 ± 0.00 | 0.00 ± 0.00 | 0.45 ± 0.70 | 1.93 ± 1.32 |
| **Firmicutes\|Bacilli\|Lactobacillales\|Lactobacillaceae\|*Limosilactobacillus*** | 4.26 ± 0.88 | 0.00 ± 0.00 | 0.00 ± 0.00 | 0.00 ± 0.00 | 0.00 ± 0.00 | 0.00 ± 0.00 | 0.95 ± 0.90 |
| **Proteobacteria\|Gammaproteobacteria\|Gammaproteobacteria Incertae Sedis\|Unknown Family\|*Acidibacter*** | 0.00 ± 0.00 | 0.00 ± 0.00 | 0.00 ± 0.00 | 0.55 ± 0.76 | 2.49 ± 2.68 | 0.24 ± 0.60 | 0.00 ± 0.00 |
| **Firmicutes\|Bacilli\|Mycoplasmatales\|Mycoplasmataceae\|*Mycoplasma*** | 0.00 ± 0.00 | 5.66 ± 2.49 | 0.00 ± 0.00 | 0.00 ± 0.00 | 0.00 ± 0.00 | 0.00 ± 0.00 | 0.00 ± 0.00 |
| **Fusobacteriota\|Fusobacteriia\|Fusobacteriales\|Fusobacteriaceae\|*Cetobacterium*** | 5.50 ± 1.15 | 0.00 ± 0.00 | 0.00 ± 0.00 | 0.00 ± 0.00 | 0.00 ± 0.00 | 0.00 ± 0.00 | 0.00 ± 0.00 |

Values are represented as mean ± SD.
