# Supplementary material for: Impact of the diet in the gut microbiota after an inter-species microbial transplantation in fish
Source: Sci Rep. 2024 Feb 18;14:4007. doi: 10.1038/s41598-024-54519-6 (PMC10874947; doi:10.1038/s41598-024-54519-6)
Supplement: Supplementary file 10 — Supplementary Table 5. [file 41598_2024_54519_MOESM10_ESM.docx]

**Table S5.** Relative abundances of phyla from the gut bacterial communities ≥ 0.5% in salmon diet, Atlantic salmon (microbiota donor), gilthead seabream previous to the intestinal microbiota transplant (GSB pre-IMT) and in gilthead seabream fed the salmon diet at 2, 7, 16 and 36 days post-IMT.

|  | **Salmon diet** | **Salmon** | **GSB pre-IMT** | **GSB 2 days**  **post-IMT** | **GSB 7 days**  **post-IMT** | **GSB 16 days**  **post-IMT** | **GSB 36 days**  **post-IMT** |
| --- | --- | --- | --- | --- | --- | --- | --- |
| **Proteobacteria** | 64.69 ± 6.09 | 93.37 ± 1.70 | 89.17 ± 3.97 | 77.18 ± 14.46 | 57.40 ± 9.53 | 43.42 ± 8.49 | 61.24 ± 12.51 |
| **Firmicutes** | 20.07 ± 2.95 | 5.87 ± 2.24 | 5.13 ± 1.06 | 6.75 ± 4.75 | 7.21 ± 7.95 | 5.14 ± 2.34 | 12.85 ± 5.98 |
| **Unassigned** | 0.91 ± 0.41 | 0.00 ± 0.00 | 5.25 ± 4.60 | 2.11 ± 3.36 | 7.10 ± 1.54 | 23.55 ± 14.43 | 2.28 ± 5.79 |
| **Actinobacteriota** | 3.69 ± 1.35 | 0.18 ± 0.32 | 0.00 ± 0.00 | 4.12 ± 3.51 | 7.41 ± 3.00 | 7.82 ± 4.12 | 10.42 ± 5.67 |
| **Bacteroidota** | 7.88 ± 1.46 | 0.18 ± 0.30 | 0.00 ± 0.00 | 1.43 ± 1.73 | 6.81 ± 4.95 | 5.11 ± 3.19 | 8.93 ± 7.05 |
| **Cyanobacteria** | 1.02 ± 0.34 | 0.00 ± 0.00 | 0.00 ± 0.00 | 3.81 ± 4.44 | 5.70 ± 3.99 | 8.11 ± 5.02 | 1.76 ± 2.66 |
| **Spirochaetota** | 0.15 ± 0.25 | 0.00 ± 0.00 | 0.45 ± 0.40 | 1.61 ± 1.49 | 6.05 ± 6.43 | 4.35 ± 3.06 | 0.31 ± 0.81 |
| **Planctomycetota** | 0.12 ± 0.20 | 0.18 ± 0.32 | 0.00 ± 0.00 | 1.09 ± 2.17 | 1.36 ± 2.19 | 0.95 ± 2.33 | 1.07 ± 2.83 |

Values are represented as mean ± SD.
