# Supplementary material for: Impact of the diet in the gut microbiota after an inter-species microbial transplantation in fish
Source: Sci Rep. 2024 Feb 18;14:4007. doi: 10.1038/s41598-024-54519-6 (PMC10874947; doi:10.1038/s41598-024-54519-6)
Supplement: Supplementary file 11 — Supplementary Table 6. [file 41598_2024_54519_MOESM11_ESM.docx]

**Table S6.** Relative abundances of genera from the gut bacterial communities ≥ 0.5% in salmon diet, Atlantic salmon (microbiota donor), gilthead seabream previous to the intestinal microbiota transplant (GSB pre-IMT) and in gilthead seabream fed the salmon diet at 2, 7, 16 and 36 days post-IMT.

|  | **Salmon diet** | **Salmon** | **GSB pre-IMT** | **GSB 2 days**  **post-IMT** | **GSB 7 days**  **post-IMT** | **GSB 16 days**  **post-IMT** | **GSB 36 days**  **post-IMT** |
| --- | --- | --- | --- | --- | --- | --- | --- |
| **Proteobacteria\|Gammaproteobacteria\|Enterobacterales\|Vibrionaceae\|*Photobacterium*** | 0.69 ± 0.26 | 7.18 ± 0.68 | 12.66 ± 3.08 | 22.92 ± 4.81 | 11.94 ± 13.85 | 4.86 ± 2.45 | 2.92 ± 3.27 |
| **Proteobacteria\|Gammaproteobacteria\|Enterobacterales\|Vibrionaceae\|*Vibrio*** | 1.72 ± 1.14 | 10.26 ± 0.38 | 56.21 ± 7.75 | 11.62 ± 5.66 | 0.63 ± 1.34 | 1.21 ± 0.96 | 0.49 ± 1.29 |
| **Proteobacteria\|Gammaproteobacteria\|Enterobacterales\|Vibrionaceae\|*Aliivibrio*** | 0.00 ± 0.00 | 57.59 ± 5.09 | 0.00 ± 0.00 | 13.11 ± 14.85 | 0.30 ± 0.74 | 0.00 ± 0.00 | 0.41 ± 0.76 |
| **Unassigned\|Unassigned\|Unassigned\|Unassigned\|Unassigned** | 0.91 ± 0.41 | 0.00 ± 0.00 | 5.25 ± 4.60 | 2.11 ± 3.36 | 7.10 ± 1.54 | 23.55 ± 14.43 | 2.28 ± 5.79 |
| **Proteobacteria\|Gammaproteobacteria\|Enterobacterales\|Enterobacteriaceae\|*Escherichia-Shigella*** | 0.91 ± 0.11 | 0.68 ± 0.62 | 0.51 ± 0.45 | 2.09 ± 0.37 | 3.44 ± 0.92 | 5.67 ± 3.24 | 19.06 ± 10.58 |
| **Proteobacteria\|Gammaproteobacteria\|Enterobacterales\|Vibrionaceae\|Unassigned** | 0.45 ± 0.55 | 13.79 ± 0.23 | 10.10 ± 1.53 | 5.92 ± 3.21 | 3.83 ± 7.11 | 3.18 ± 5.10 | 1.44 ± 3.10 |
| **Proteobacteria\|Gammaproteobacteria\|Pseudomonadales\|Moraxellaceae\|*Acinetobacter*** | 1.46 ± 0.10 | 0.00 ± 0.00 | 0.00 ± 0.00 | 2.30 ± 3.36 | 3.03 ± 1.95 | 7.31 ± 3.26 | 7.70 ± 3.69 |
| **Cyanobacteria\|Cyanobacteriia\|Synechococcales\|Cyanobiaceae\|*Synechococcus* CC9902** | 0.00 ± 0.00 | 0.00 ± 0.00 | 0.00 ± 0.00 | 2.74 ± 3.19 | 3.83 ± 3.12 | 5.39 ± 3.09 | 1.45 ± 2.73 |
| **Proteobacteria\|Gammaproteobacteria\|Enterobacterales\|Vibrionaceae\|*Catenococcus*** | 0.92 ± 0.30 | 0.00 ± 0.00 | 6.73 ± 1.08 | 5.55 ± 2.04 | 0.82 ± 1.29 | 1.60 ± 1.37 | 2.00 ± 3.47 |
| **Spirochaetota\|Brevinematia\|Brevinematales\|Brevinemataceae\|*Brevinema*** | 0.15 ± 0.24 | 0.00 ± 0.00 | 0.45 ± 0.40 | 1.61 ± 1.49 | 5.83 ± 6.63 | 4.35 ± 3.06 | 0.31 ± 0.81 |
| **Proteobacteria\|Gammaproteobacteria\|Burkholderiales\|Comamonadaceae\|Unassigned** | 0.15 ± 0.25 | 0.00 ± 0.00 | 0.00 ± 0.00 | 0.00 ± 0.00 | 8.57 ± 11.1 | 0.33 ± 0.81 | 2.01 ± 2.91 |
| **Actinobacteriota\|Actinobacteria\|Corynebacteriales\|Corynebacteriaceae\|*Corynebacterium*** | 0.10 ± 0.17 | 0.00 ± 0.00 | 0.00 ± 0.00 | 2.39 ± 2.76 | 1.76 ± 1.18 | 3.24 ± 2.66 | 3.12 ± 3.04 |
| **Firmicutes\|Bacilli\|Staphylococcales\|Staphylococcaceae\|*Staphylococcus*** | 0.41 ± 0.01 | 0.21 ± 0.37 | 0.00 ± 0.00 | 1.85 ± 1.73 | 1.18 ± 1.27 | 2.03 ± 1.73 | 3.83 ± 3.22 |
| **Proteobacteria\|Gammaproteobacteria\|Pseudomonadales\|Pseudomonadaceae\|*Pseudomonas*** | 1.28 ± 0.16 | 0.00 ± 0.00 | 0.00 ± 0.00 | 2.69 ± 3.27 | 0.84 ± 1.35 | 0.64 ± 0.99 | 2.24 ± 3.17 |
| **Firmicutes\|Bacilli\|Lactobacillales\|Carnobacteriaceae\|*Alloiococcus*** | 0.00 ± 0.00 | 0.00 ± 0.00 | 0.00 ± 0.00 | 0.00 ± 0.00 | 1.10 ± 2.69 | 0.00 ± 0.00 | 4.62 ± 5.07 |
| **Proteobacteria\|Alphaproteobacteria\|Rhizobiales\|Xanthobacteraceae\|Unassigned** | 0.11 ± 0.19 | 0.00 ± 0.00 | 0.00 ± 0.00 | 0.00 ± 0.00 | 2.13 ± 2.40 | 0.23 ± 0.56 | 3.10 ± 3.77 |
| **Bacteroidota\|Bacteroidia\|Chitinophagales\|Chitinophagaceae\|*Vibrionimonas*** | 0.35 ± 0.31 | 0.00 ± 0.00 | 0.00 ± 0.00 | 0.00 ± 0.00 | 0.00 ± 0.00 | 0.49 ± 0.77 | 4.44 ± 5.93 |
| **Actinobacteriota\|Actinobacteria\|Propionibacteriales\|Propionibacteriaceae\|*Cutibacterium*** | 0.11 ± 0.19 | 0.00 ± 0.00 | 0.00 ± 0.00 | 0.51 ± 0.60 | 1.06 ± 1.16 | 1.28 ± 0.74 | 2.05 ± 2.13 |
| **Proteobacteria\|Gammaproteobacteria\|Pseudomonadales\|Halomonadaceae\|*Halomonas*** | 8.00 ± 0.88 | 0.00 ± 0.00 | 0.00 ± 0.00 | 0.00 ± 0.00 | 0.37 ± 0.90 | 0.64 ± 1.57 | 0.00 ± 0.00 |
| **Proteobacteria\|Gammaproteobacteria\|Pseudomonadales\|Halomonadaceae\|*Chromohalobacter*** | 8.28 ± 0.64 | 0.28 ± 0.49 | 0.00 ± 0.00 | 0.00 ± 0.00 | 0.00 ± 0.00 | 0.00 ± 0.00 | 0.00 ± 0.00 |
| **Firmicutes\|Bacilli\|Mycoplasmatales\|Mycoplasmataceae\|*Mycoplasma*** | 0.00 ± 0.00 | 5.66 ± 2.49 | 0.00 ± 0.00 | 0.00 ± 0.00 | 1.24 ± 2.40 | 0.00 ± 0.00 | 0.00 ± 0.00 |
| **Bacteroidota\|Bacteroidia\|Chitinophagales\|Chitinophagaceae\|*Asinibacterium*** | 0.00 ± 0.00 | 0.00 ± 0.00 | 0.00 ± 0.00 | 0.00 ± 0.00 | 1.67 ± 2.06 | 0.00 ± 0.00 | 2.02 ± 2.95 |
| **Proteobacteria\|Gammaproteobacteria\|Enterobacterales\|Idiomarinaceae\|*Idiomarina*** | 6.95 ± 2.13 | 0.23 ± 0.40 | 0.00 ± 0.00 | 0.24 ± 0.47 | 0.00 ± 0.00 | 0.00 ± 0.00 | 0.00 ± 0.00 |
| **Proteobacteria\|Alphaproteobacteria\|Sphingomonadales\|Sphingomonadaceae\|*Sphingomonas*** | 0.11 ± 0.19 | 0.00 ± 0.00 | 0.00 ± 0.00 | 0.66 ± 0.77 | 0.00 ± 0.00 | 0.61 ± 1.50 | 2.16 ± 4.30 |
| **Firmicutes\|Clostridia\|Clostridiales\|Clostridiaceae\|Unassigned** | 0.11 ± 0.18 | 0.00 ± 0.00 | 4.78 ± 0.83 | 0.00 ± 0.00 | 1.18 ± 2.89 | 0.00 ± 0.00 | 0.00 ± 0.00 |
| **Bacteroidota\|Bacteroidia\|Chitinophagales\|Chitinophagaceae\|*Heliimonas*** | 0.00 ± 0.00 | 0.00 ± 0.00 | 0.00 ± 0.00 | 0.00 ± 0.00 | 2.89 ± 3.23 | 0.00 ± 0.00 | 0.61 ± 1.62 |
| **Proteobacteria\|Alphaproteobacteria\|Rhizobiales\|Xanthobacteraceae\|*Bradyrhizobium*** | 0.00 ± 0.00 | 0.00 ± 0.00 | 0.00 ± 0.00 | 0.00 ± 0.00 | 3.03 ± 3.59 | 0.00 ± 0.00 | 0.17 ± 0.45 |
| **Proteobacteria\|Gammaproteobacteria\|Unassigned\|Unassigned\|Unassigned** | 0.29 ± 0.37 | 0.18 ± 0.32 | 0.00 ± 0.00 | 1.04 ± 1.28 | 0.62 ± 0.97 | 0.64 ± 0.87 | 0.65 ± 1.54 |
| **Firmicutes\|Bacilli\|Bacillales\|Bacillaceae\|*Bacillus*** | 0.50 ± 0.05 | 0.00 ± 0.00 | 0.35 ± 0.34 | 0.41 ± 0.82 | 0.20 ± 0.48 | 0.00 ± 0.00 | 1.75 ± 4.64 |
| **Cyanobacteria\|Cyanobacteriia\|Synechococcales\|Cyanobiaceae\|*Cyanobium* PCC-6307** | 0.00 ± 0.00 | 0.00 ± 0.00 | 0.00 ± 0.00 | 1.08 ± 1.25 | 0.76 ± 1.30 | 1.36 ± 1.71 | 0.00 ± 0.00 |
| **Actinobacteriota\|Actinobacteria\|Corynebacteriales\|Corynebacteriaceae\|*Turicella*** | 0.00 ± 0.00 | 0.00 ± 0.00 | 0.00 ± 0.00 | 0.00 ± 0.00 | 0.00 ± 0.00 | 0.00 ± 0.00 | 2.30 ± 3.41 |
| **Proteobacteria\|Gammaproteobacteria\|Enterobacterales\|Pseudoalteromonadaceae\|*Pseudoalteromonas*** | 4.31 ± 0.96 | 1.03 ± 0.44 | 0.00 ± 0.00 | 0.00 ± 0.00 | 0.00 ± 0.00 | 0.00 ± 0.00 | 0.00 ± 0.00 |

Values are represented as mean ± SD.
